# *Pseudomonas aeruginosa* pulmonary infection results in S100A8/A9-dependent cardiac dysfunction

**DOI:** 10.1371/journal.ppat.1011573

**Published:** 2023-08-25

**Authors:** Naresh Kumar, Matthew J. Pestrak, Qian Wu, Omar Santiagonunez Ahumada, Sheri Dellos-Nolan, Noushin Saljoughian, Rajni Kant Shukla, Cortney F. Mitchem, Prabhakara R. Nagareddy, Latha P. Ganesan, Lafuse P. William, Daniel J. Wozniak, Murugesan V. S. Rajaram

**Affiliations:** 1 Department of Microbial Infection and Immunity, College of Medicine, The Ohio State University, Wexner Medical Center, Columbus, Ohio, United States of America; 2 Department of Microbiology, College of Medicine, The Ohio State University, Wexner Medical Center, Columbus, Ohio, United States of America; 3 Department of Surgery, College of Medicine, The Ohio State University, Wexner Medical Center, Columbus, Ohio, United States of America; 4 Department of Internal Medicine, College of Medicine, The Ohio State University, Wexner Medical Center, Columbus, Ohio, United States of America; university of washington, UNITED STATES

## Abstract

*Pseudomonas aeruginosa* (*P*.*a*.) infection accounts for nearly 20% of all cases of hospital acquired pneumonia with mortality rates >30%. *P*.*a*. infection induces a robust inflammatory response, which ideally enhances bacterial clearance. Unfortunately, excessive inflammation can also have negative effects, and often leads to cardiac dysfunction with associated morbidity and mortality. However, it remains unclear how *P*.*a*. lung infection causes cardiac dysfunction. Using a murine pneumonia model, we found that *P*.*a*. infection of the lungs led to severe cardiac left ventricular dysfunction and electrical abnormalities. More specifically, we found that neutrophil recruitment and release of S100A8/A9 in the lungs activates the TLR4/RAGE signaling pathways, which in turn enhance systemic inflammation and subsequent cardiac dysfunction. Paradoxically, global deletion of S100A8/A9 did not improve but aggravated cardiac dysfunction and mortality likely due to uncontrolled bacterial burden in the lungs and heart. Our results indicate that *P*.*a*. infection induced release of S100A8/9 is double-edged, providing increased risk for cardiac dysfunction yet limiting *P*.*a*. growth.

## Author summary

Over 5 million people develop pneumonia annually in the United States and is the 8^th^ leading cause of death worldwide. Surprisingly, up to 30% of patients admitted to the hospital for community acquired pneumonia develop cardiovascular complications but the mechanisms are not clearly understood. *Pseudomonas aeruginosa (P.a.)* infection is very common in hospital settings and accounts for up to 20% of all cases of hospital-acquired pneumonia with a mortality rate of ~30%. *P.a.* infection and severe lung and systemic inflammation increases levels of S100A8/9 (calprotectin) in the blood stream. Systemic inflammation or dissemination of pathogens into heart tissue can induce cardiac inflammation, cardiomyocyte death and cardiac dysfunction. We found that *P.a.* infection increases infiltration of inflammatory immune cells in the heart, leading to arrhythmia and left ventricular dysfunction. Our study provides evidence that S100A8/9 provides protection against bacterial infection yet when its production is uncontrolled it activates host immune responses leading to severe cardiovascular complications.

## Introduction

Persistent lung infection often develops into severe pneumonia and contributes to increased mortality [1]. Over 5 million people develop pneumonia annually in the United States alone and is the 8^th^ leading cause of death worldwide. Furthermore, secondary infections in severely sick patients in intensive care units (ICU) remain a common and persistent problem in health care [2]. Surprisingly, up to 30% of patients admitted to the hospital for community acquitted pneumonia develop cardiovascular complications [3] but the mechanisms are not clearly understood. Indeed, adverse cardiac complications such as myocardial infarction, arrhythmia, left ventricular dysfunction, and heart failure are the major contributing factors to increased mortality in hospitalized people with pneumonia [4].

*Pseudomonas aeruginosa* (*P*.*a*.) is the most common nosocomial infectious agent present in the hospital settings [5] and known to cause multi organ dysfunction syndrome (MODS) in patients admitted to the ICU [6]. *P*.*a*. is a gram-negative bacterium loaded with various arsenals to activate, modulate, and destroy the host immune defense system. Specifically, *P*.*a*. infections account for up to 20% of all cases of hospital-acquired pneumonia with a mortality rate of ~30%. Also, *P*.*a*. infections are very difficult to treat since this bacterium is naturally resistant to many routine antibiotics [7]. Furthermore, *P*.*a*. infection is a major driver of increased mortality in people with chronic lung diseases such as cystic fibrosis, COPD, and bronchiectasis [8]. *P*.*a*.*-*mediated release of bacterial pathogen-associated molecular patterns (PAMPs), host inflammatory mediators and damage-associated molecular patterns (DAMPs) into the circulation increases extravasation of leukocytes into the organs leading to secondary complications including cardiac dysfunction and associated mortality [2,9]. It is well recognized that cardiac complications exacerbates both infectious and non-infectious MODS [10]. Persistent lung infection and inflammation can cause tissue damage and malfunction including cardiac inotropy. Additionally, circulating endotoxins and other PAMPs/DAMPs have the capacity to activate platelets, generating a procoagulant state that facilitate acute coronary symptoms and activation of the sympathetic nervous system. These events invariably lead to increased heart rate and vascular resistance that in turn lower cardiac output and coronary perfusion of the heart [3,11,12]. However, the molecular mechanisms of cardiac dysfunction caused by *P*.*a*. infection is not well-understood.

Like other organs, heart tissue is composed of several cell types including fibroblasts, cardiomyocytes, macrophages and other immune cells [13]. Systemic inflammation or dissemination of pathogens into heart tissue can induce cardiomyocyte death, which is cleared by resident macrophages that subsequently produce IL-10 and TGF-β. Notably, the activation of fibroblasts by IL-10 and TFG-β enhances secretion of collagen and extracellular matrix (ECM) to maintain the heart tissue architecture [14,15]. However, uncontrolled cardiac inflammation and fibroblast activation may promote cardiac fibrosis, leading to structural remodeling of myocardium and eventually cardiac hypertrophy [16]. Electrical remodeling is the manifestation of structural remodeling and is associated with alterations of ion channels, EC-coupling (Ca^2+^ cycling) and intracellular gap junctions [17]. As a result, cardiac electrical dysfunction, cardiac hypertrophy, and cardiac arrhythmias occur in the heart and disturb normal cardiac function. Moreover, several pathogens upregulate the sympathetic nervous system which leads to increased contractility and decreased cardiac output, creating a state of cardiac contractile dysfunction [3].

*P*.*a*. infection enhances neutrophil recruitment and induces a robust immune response, leading to release of reactive oxygen species, various proteases, and NETosis [18,19]. Previous studies have demonstrated that ~45% of the neutrophil’s cytoplasmic protein content are S100A8 and S100/A9, that are rapidly released in response to infection, inflammation, and other events of cellular stress [20,21]. S100A8 and S100A9 are members of the S100 protein family and are constitutively expressed in early myeloid lineage cells, yet tissue resident macrophages lack their expression [22]. The S100A8/A9 proteins are known for their role in chemotaxis, phagocytosis, respiratory burst, and exocytosis of phagocytic cells [23–25]. Furthermore, Zaia et al., demonstrated that S100A8/A9 attenuates bacterial adherence and invasion [26] by sequestrating essential metals such as Zn^2+^ and Mn^2+^, which are required for bacterial growth [27,28]. Additionally, S100A8A/9 enhances recruitment of neutrophils and macrophages at the site of infection and increases the phagocytic property of neutrophils [29,30]. Taken together, S100A8/A9 performs a dual function by inhibiting bacterial growth and recruitment/ activation of phagocytic cells, the latter function attributed to its binding with TLR4 and RAGE (receptor for advance glycation product) receptors [31]. However, elevated, and persistent production of S100A8/9 can cause severe damage to heart tissue during infection [32]. John *et al*., showed that *S100a9*^*-/-*^ mice are protected from LPS-induced cardiac dysfunction [32]. Together, these studies suggests that S100A8/A9 is detrimental for the pathogenesis of *P*.*a*. lung infection and cardiac dysfunction but the underlying signaling mechanisms are not clear.

In the current study, we utilized an *in vivo* murine infection model to investigate the host response to *P*.*a*. infection and the role of S100A8/A9 in the regulation of heart function during infection. We observed that *P*.*a*. infection increases mortality by causing severe cardiac electrical dysfunction and arrhythmia, and that S100A8/A9 released by neutrophils is essential for providing protection against the *P*.*a*. infection. However, uncontrolled production of S100A8/A9 hyper-activates the innate immune system and promotes cardiac dysfunction and mortality.

## Results

### *P*.*a*. infection colonizes the lungs of mice and enhances mortality

**Fig 1:**
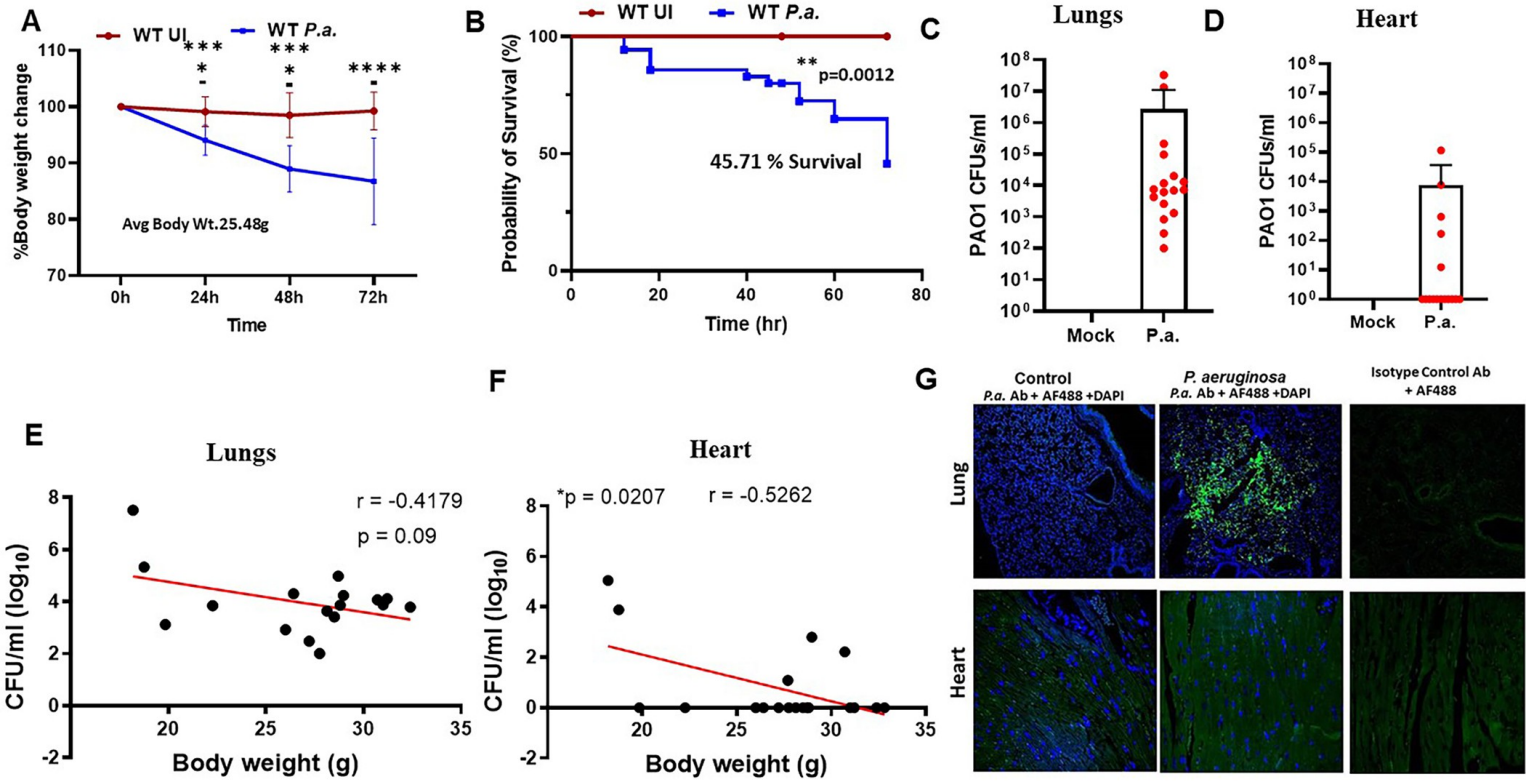
*P*. *aeruginosa* lung infection of mice enhances morbidity and mortality. C57BL/6 mice were intranasally infected with sequential doses of *P*. *aeruginosa* strain PAO1 (1x10^8^ CFU and after 24h 1x10^7^ CFU and body weight and survival of the mice monitored for 72h. (**A**) Percent weight loss and (**B**) survival rate was calculated every 24h following infection. Data shown are accumulative from 5 different experiments (n = 16 control mock-infected mice and 35 *P*.*a*.-infected mice) mean ± SD **p<0.*05;* ***p<0.005; ****p<0.*0005*. At the end of the study period, surviving mice were euthanized, lungs and hearts were harvested, homogenized and bacterial burden quantified. Graphs shown in (**C**) lungs and (**D**) heart are the CFU burden in the organs. Based on the bacterial load in the lung we stratified the mice into mild and severely infected groups. Graphs E and F shows the correlation of age and bacterial CFU. (**G**) Lung and heart cross-sections from uninfected controls (first column) and *P*.*a*. infected (middle column) were stained with α-*P*. *aeruginosa* antibody and Alexa 488 conjugated secondary antibody or with an isotype control (last column). The sections were examined for *P*.*a*. by confocal microscopy. Images shown are representative of lungs (top panel) and hearts (bottom panel) from five control uninfected mice and five *P*.*a*. *infected* mice.

*P*.*a*. is known to cause lung infections in humans which can result in severe pneumonia and leading to heart failure and death [33]. To establish a model that parallels human disease, we used C57BL/6 WT mice infected by the intranasal route with *P*.*a*. strain PAO1. Significant weight loss and morbidity was observed in all *P*.*a*. infected mice compared to the uninfected control mice (Fig 1A). Further, Kaplan-Meier survival analyses showed that the *P*.*a*. infection significantly decreased survival of infected mice (45.71%) in comparison to the control mice (Fig 1B). Interestingly, younger mice (8–10 weeks) with a body weight of 18–22 grams showed extremely poor survival rate compared to older mice (12–15 weeks) weighing 22–30 grams. Bacterial colonization was apparent in all infected mice, yet bacterial burden varied from 10^2^ to 10^8^ /lung suggesting that some mice with higher body weight are capable of controlling *P*.*a*. infection (Fig 1C). We have used total of 35 mice for *P*.*a*. infections, and most of the mice continued to lose weight and had increased CFUs in the lungs. However, some of the infected mice started gaining weight after 48 hours, and this weight gain correlated with decreased *P*.*a*. burden in the lungs. Notably, some mice with severe lung infections also had *P*.*a*. detected in the heart. Out of 20 mice, the hearts of 5 mice were colonized by *P*.*a*., and the infection burden varied from 10^3^−10^5^ CFU (Fig 1D). Since we found variable levels of organ *P*.*a*. colonization, we correlated the age/ body weight with bacterial CFU. The bacterial load in the lungs and hearts was plotted against mice body weight, which R-value of lung and heart CFUs at -0.4179 and -0.5262, respectively. Thus, there is a negative correlation between bacterial load in both lungs and hearts and mice body weight (Fig 1E and 1F). Immunofluorescence microscopy analysis using anti-*P*.*a*. serum supported the CFU data showing bacterial colonization in the lungs, but not in the heart (the latter likely due to limitation of the staining; Fig 1G). Additionally, we examined immune cell infiltration and apoptosis by probing the heart section with CD45 antibody and TUNEL assay respectively. These results revealed that *P*.*a*. infection increased the accumulation of CD45 positive cells and TUNEL positive cells in the hearts (S1A–S1D Fig). To confirm cardiac damage upon *P*.*a*. infection, cardiac troponin I levels in the serum of uninfected and *P*.*a*. infected animals was measured and showed that *P*.*a*. infection increases serum troponin levels (S1E Fig). Together, these data indicating that infection causes severe cardiac inflammation and damage. Thus, our results suggest that bacterial dissemination to the heart occurs only during severe *P*.*a*. lung infections.

### *P*.*a*. infection causes abnormalities in cardiac electrical activity and heart function

Lung infections often progresses to severe multi-organ dysfunction syndrome, due to bacteremia and spread to other vital organs. We and others have demonstrated that lung infections caused by bacteria and viruses can disseminate into the heart and cause cardiac electrical dysfunction and compromised left ventricular function [34,35,36]. Thus, we examined whether *P*. *a*. infection causes cardiac dysfunction in severely infected animals with and without *P*.*a*. dissemination in the heart tissue.

**Fig 2:**
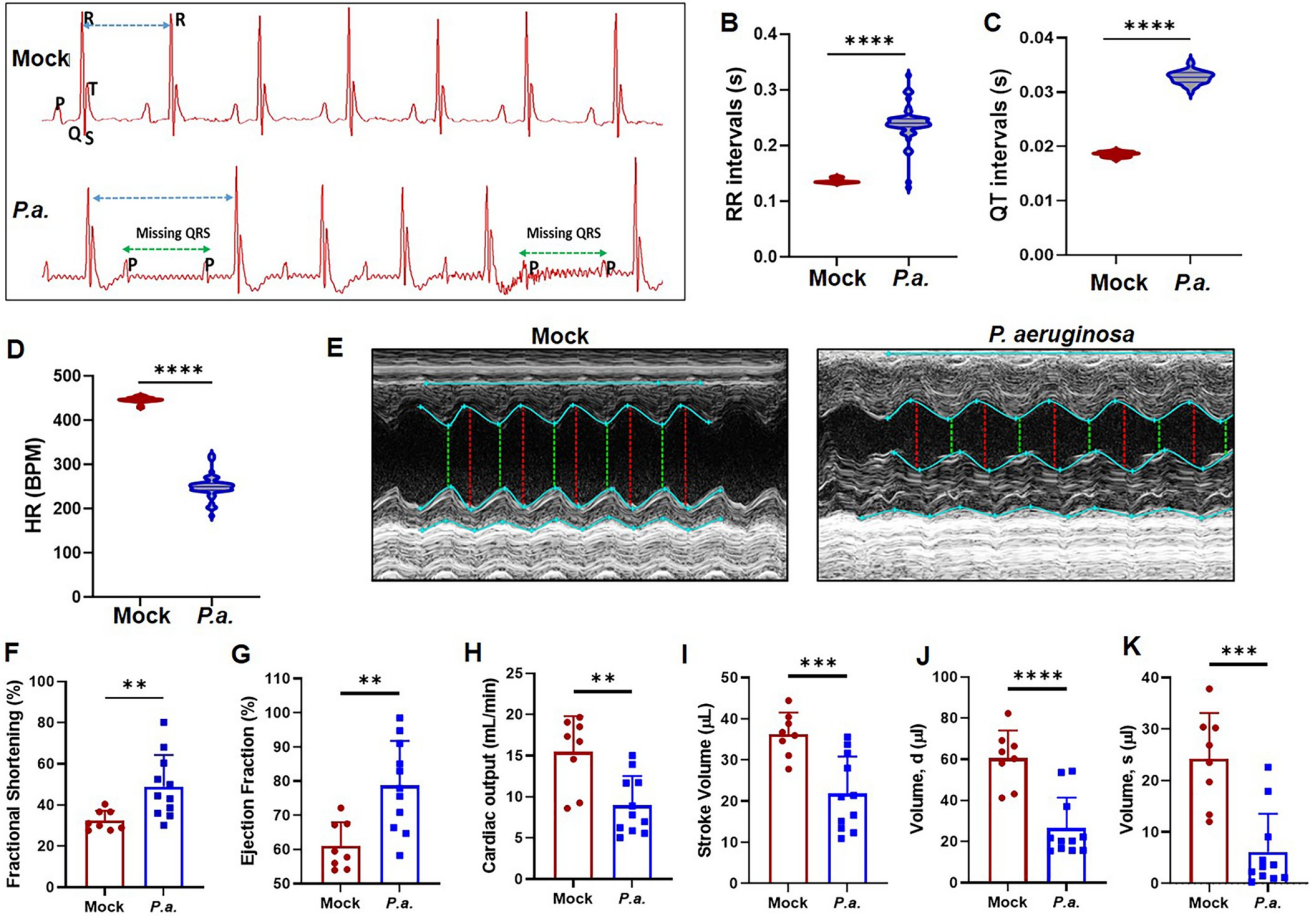
*P*. *aeruginosa* pulmonary infection results in cardiac electrical- and left ventricular- dysfunction. C57BL/6 mice were infected via intranasal instillation with *P*.*a*. as described in Fig 1. After 48h, cardiac electrical activity and heart function were measured by electrocardiography (ECG) and echocardiography respectively. (**A**) image shows a representative ECG trace from control mock uninfected mice (top panel) and *P*.*a*. infected mice (bottom panel). The blue arrows indicate the RR intervals, and the green arrows indicate missing QRS in infected mice. ECG traces were analyzed using Lab Chart 8 Pro (AD Instruments) and the RR intervals (**B**), QT intervals (**C**) and heart rate (**D**) were calculated. To assess heart function *in vivo*, 2D-echocardiography (Vevo 2100, Visualsonics) was performed in control mock infected mice and *P*.*a*. infected mice at 48h post infection. (**E**) Representative images of cardiac patches from control mock and *P*.*a*. infected mice. At least 3 M-mode echocardiogram measurements from each mouse were used to determine the fractional shortening (**F**), % ejection fraction (**G**), cardiac output (**H**), stroke volume (**I**), left ventricular volume during diastole (**J**) and systole (**K**). Data shown are the mean ± SD of accumulative data from three independent experiments (N = 11), **p<0.*005;* ***p<0.0005; ****p<0.*00005*.

To assess cardiac electrical activity in *P*.*a*. infected animals, we performed an electrocardiogram (ECG) and analyzed the data using ADInstruments Lab Chart software. Compared to uninfected control mice, the severely infected animals showed an abnormal ECG pattern with missing QRS peak and irregular RR intervals (Fig 2A), suggesting that these mice experience second degree atrioventricular (AV) block in which the conduction of the atrial impulse through the AV node and/ or His bundle is delayed or blocked. Next, we calculated the RR intervals, QT intervals, and heart rate from the recorded EKG tracing (5 min). We also found increased RR intervals (Fig 2B) in *P*.*a*. severely infected mice. The QT interval measures the duration of QRS complex and the end of the T wave. We also observed an extended interval between heart contraction and relaxation (prolongation of QT intervals) in the *P*.*a*. infected mice compared to the uninfected control mice (Fig 2C). Since the RR and QT intervals of the *P*.*a*. infected mice were increased, there was a corresponding decrease in the heart rate (Fig 2D). Notably, the mildly infected mice showed a different pattern of cardiac electrical activity with no significant differences in RR intervals, QT intervals, or heart rate.

Defective conduction of electrical activity in the heart is correlated with decreased pumping efficiency. Thus, to assess *in vivo* cardiac function in these animals, we performed an echocardiography. The cardiac patch shown in Fig 2E (right panel) is a representative of 11 severely infected mice. The LV echocardiogram of *P*.*a*. infected mice showed significant thickening of both anterior and posterior heart walls when compared to uninfected control mice (Figs 2E and S2A–S2D). As a result, we observed significantly increased fractional shortening (FS), ejection fraction (EF), significantly reduced cardiac output, stroke volume, diastolic volume, and systolic volume of the LV during *P*.*a*. infection (Fig 2F–2K). Notably, although bacterial dissemination into the heart was limited (5/20 mice, Fig 1D), our results showed both irregular cardiac electrical activity and defects in left ventricular function in eleven mice with severe *P*.*a*. lung infections, even though the hearts of these mice were not colonized. Thus, the source for this cardiac dysfunction may be due to inflammatory mediators released from infected cells in the lungs, or bacterial products released into the circulation. Collectively, these data strongly indicate that *P*.*a*. infection impedes normal cardiac function, which contributes to mortality regardless of bacterial dissemination into the heart.

Next, we tested whether antibiotic treatment reverses *P*.*a*. infection induced cardiac dysfunction. We infected mice with *P.a.* and after 6 hours, one group was left untreated, and another group was treated with tobramycin (30mg/kg) and treatment continued for every 24 hours. Tobramycin enhanced survival of mice (3 out of 10) with reduced bacterial load in the lung and heart (S3A, S3B and S3C Fig). However, those mice that did survive still showed a severe defect in cardiac output as evidenced by an increase in ejection fraction, fractional shortening (S3D and S3E Fig) and reduced stroke volume (S3G Fig). Together, these data indicate that *P*.*a*. mediated inflammation persists for longer period and cause cardiac dysfunction, even after antibiotic treatment.

### *P*.*a*. infection enhances immune cell expansion in the heart

**Fig 3:**
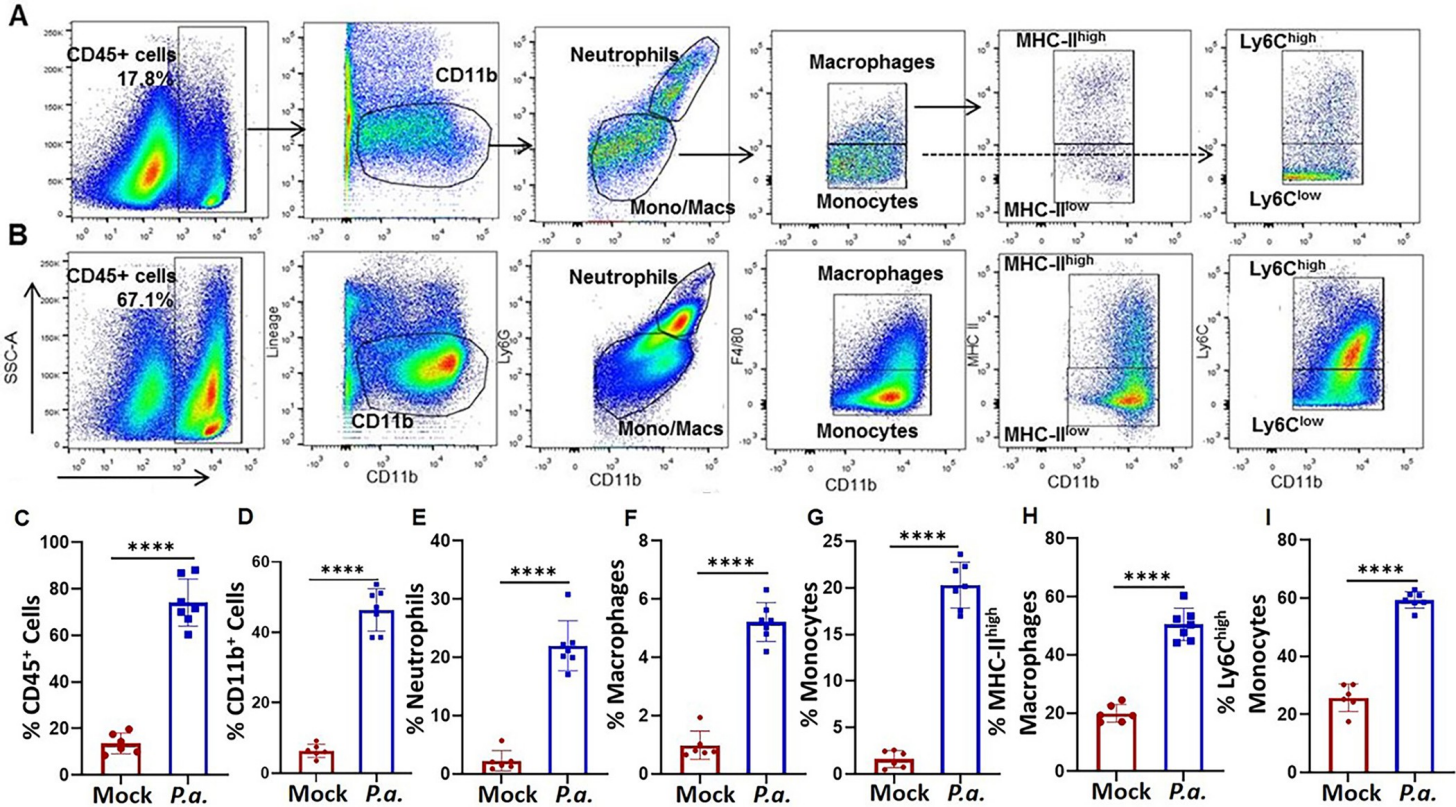
*P*. *aeruginosa* pulmonary infection enhances infiltration of cardiac myeloid cell into the hearts of mice. Hearts from *P*.*a*. infected (N = 7) and control mock infected (N = 3) C57BL/6 mice were harvested, perfused, and digested to obtain single cell suspensions. After red blood cell lysis, myeloid cells were stained and analyzed by multi-color flow cytometry. Gating strategies are shown for different cell populations from control mock infected (**A**) and *P*.*a*. infected (**B**) mice, representative of 7 mice from three independent experiments. The percentages of different cell population such as CD45^+^ cells (**C**), CD11b^+^ cells (**D**), neutrophils (**E**), macrophages (**F**),monocytes (**G**) MHC-II^high^ macrophages (**H**) and Ly6C^high^ monocytes (**I**) were determined. These data shown in the graphs are cumulative data from three experiments with surviving mice (mean ± SD; ***p<0.*0005)*.

Since cardiac inflammation is a hallmark for cardiac-electrical abnormalities, hypertrophy, and fibrosis, we next investigated the phenotype of cardiac immune cells after *P*.*a*. infection [37,38]. Cells were isolated from the hearts of control and *P*.*a*. infected animals, digested, and analyzed by multicolor flow cytometry with myeloid cell markers. Lymphocytes were identified using the pan-leukocyte marker CD45, and further gated based on the markers for specific cell types (dot plots in Fig 3A and 3B are the gating strategies used for cardiac myeloid cell phenotyping). As expected, we observed increased infiltration of CD45^+^ myeloid cells in the heart tissue during *P*.*a*. infection (Fig 3C). Consistent with the CD45 staining, there was a 5-fold increase in CD11b^+^ cells in the heart tissue following infection, indicating an influx or expansion of myeloid cells (Fig 3D). To differentiate myeloid cell subsets within the CD11b^+^ population, we used Ly6G as a marker for neutrophils and F4/80 to identify macrophages. Monocytes were identified as being F4/80^-^ and Ly6C^+^. We observed an increased number of Ly6G neutrophils, F4/80^+^ macrophages and Ly6C^+^ monocytes in the heart during infection (Fig 3E–3G). Finally, we quantified resident cardiac macrophages based on MHC-II expression and Ly6C expression. There was a large population of MHC-II^high^ macrophages and Ly6C ^high^ monocyte population in the heart during infection (Fig 3H and 3I), which suggests the recruited monocytes differentiate into an inflammatory macrophage population. Based on these data, we conclude that *P*.*a*. infection enhances recruitment of inflammatory immune cells to the heart. Given the known association of excessive inflammation with increased fibrosis, it is likely that the accumulation of these cells in the heart during *P*.*a*. infection contributes to cardiac damage and dysfunction.

*P*.*a*. infection is associated with inflammation and an increase in pro-inflammatory cytokine production [39–42]. Since we observed an abundance of inflammatory macrophages in the heart during infection, we next examined expression of key cytokine and chemokine genes in cardiac resident macrophages. Total RNA was isolated from tissue resident cardiac macrophages (CD11b^+^ and F4/80^+^) and qRT-PCR used to determine the expression of CCR2, IL-1β, TGF-β and IL-10 mRNA (S4 Fig). Increased mRNA levels of these four inflammatory mediators were observed, which suggests that the cardiac macrophages are heterogeneous in nature because they show increased expression of both pro- and anti-inflammatory cytokine genes. Our results suggest that excessive infiltration of myeloid cells into the heart tissue may dysregulates cardiac resident macrophage activity and cardiomyocyte death. This, causes tissue damage leading to altered cardiac electric activity and decreased heart function.

### *P*.*a*. infection causes the loss of AMs from the alveolar space and increased the infiltration of circulating monocytes and neutrophils in the lungs

**Fig 4:**
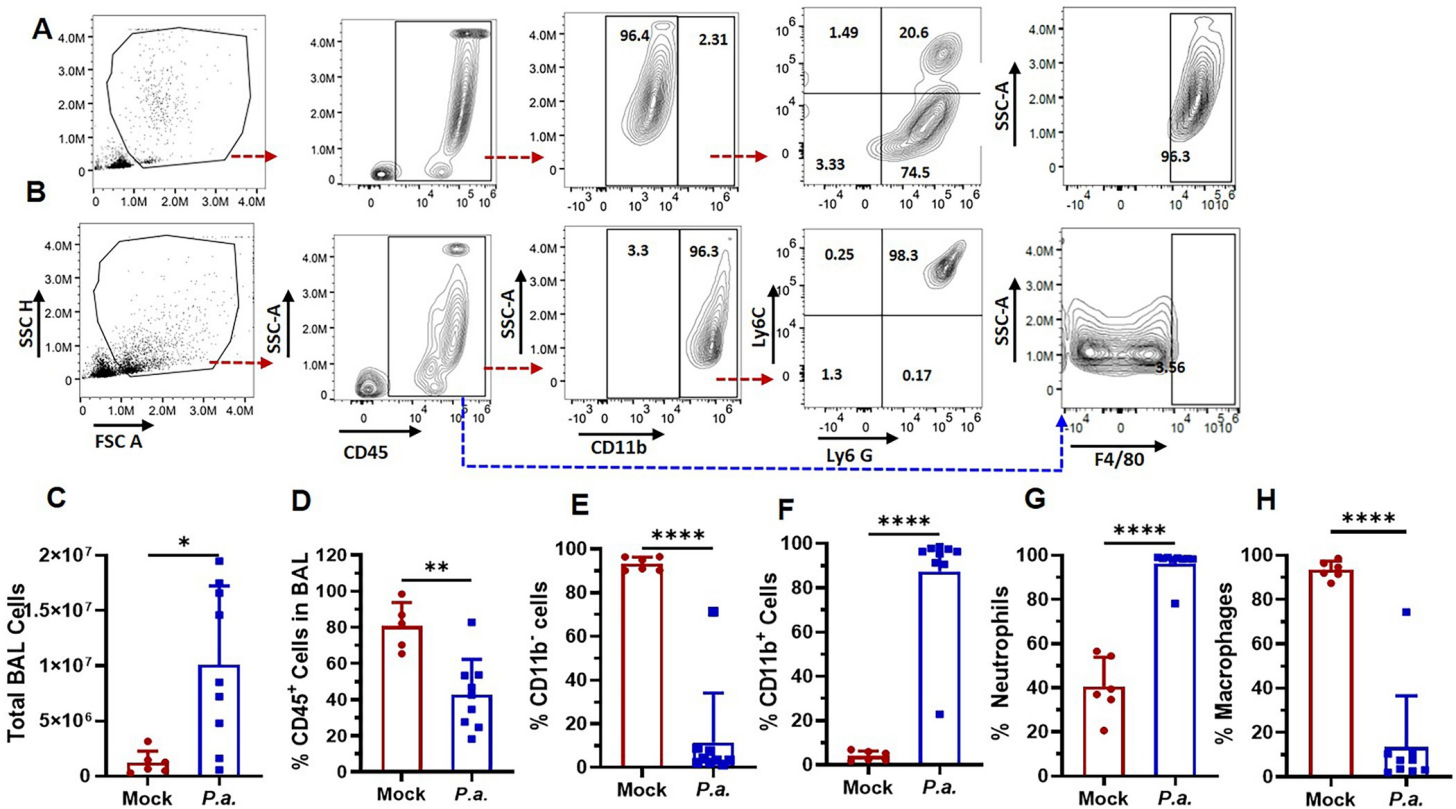
*P*.*a*. infection increases neutrophil infiltration and loss of alveolar macrophage in the lungs. C57BL/6 mice were infected via intranasal instillation with *P*.*a*. as described in Fig 1. After 48h, mice were euthanized, and alveolar lining fluid was obtained by bronchoalveolar lavage (BAL) by washing the lungs 6 times with warm sterile endotoxin free saline. Cells in the BAL fluid were collected by centrifugation, stained with CD45, CD11b, F4/80, Ly6C and Ly6G antibodies, and analyzed by multicolor flow cytometry. Gating strategies for different myeloid cell populations are shown for control mock uninfected mice (**A**) and *P*.*a*. infected (**B**). Total BAL cells were enumerated using an automated cell counter (**C**). The graph show the percentage of CD45 positive cells (**D**), CD11b negative cells (**E**) CD11b positive cells (**F**), neutrophils (**G**) and macrophages (**H**). These data are cumulative from 6 mock uninfected mice and 9 *P*.*a*. infected mice from three independent experiments (mean ± SD, *p<0.*05;* **p<0.005; *** p<0.0005; ****p<0.00005*)*.

Since, we did not observe *P*.*a*. in the hearts of most infected mice, we tested the hypothesis that systemic inflammation adversely impacts cardiac function [43–45]. We therefore examined lung inflammation by infecting mice with *P*.*a*., harvesting broncho alveolar lavage fluid (BALF), and analyzing immune cell populations by flow cytometry. Single cells were first selected from the entire population (Fig 4A and 4B), and selected lymphocytes by gated for CD45^+^ cells. *P*.*a*. infection increased the total cells in the BALF (Fig 4C), however, the number of CD45+ cells were decreased by *P*.*a*. infection (Fig 4D). Alveolar macrophages (AMs) in mock infected mice are 96% CD11c^+^ CD11b^-^ with only a small percentage being CD11c^+^ CD11b^+^ [46]. In the *P*.*a*. infected mice, we observed a significantly decreased percentage of CD11b negative cells, which are AMs (Fig 4E) and total macrophages (F4/80 positive cells) (Fig 4H), suggesting *P*.*a*. infection causes AM death. *P*.*a*. infection alsocausses increased the accumulation of CD11b^+^ cells (Fig 4F) in the BALF, which are more likely the infiltrated neutrophils (Fig 4G).

Furthermore, we also found a significantly increased F4/80^+^ population which mainly represents circulating monocyte-derived macrophages (Fig 4H) Thus, our data show that *P*.*a*. infection causes the loss of AMs from the alveolar space and increased infiltration of circulating monocytes and neutrophils.

### *P*.*a*. infection increases inflammatory cytokine levels in BALF and serum

**Fig 5:**
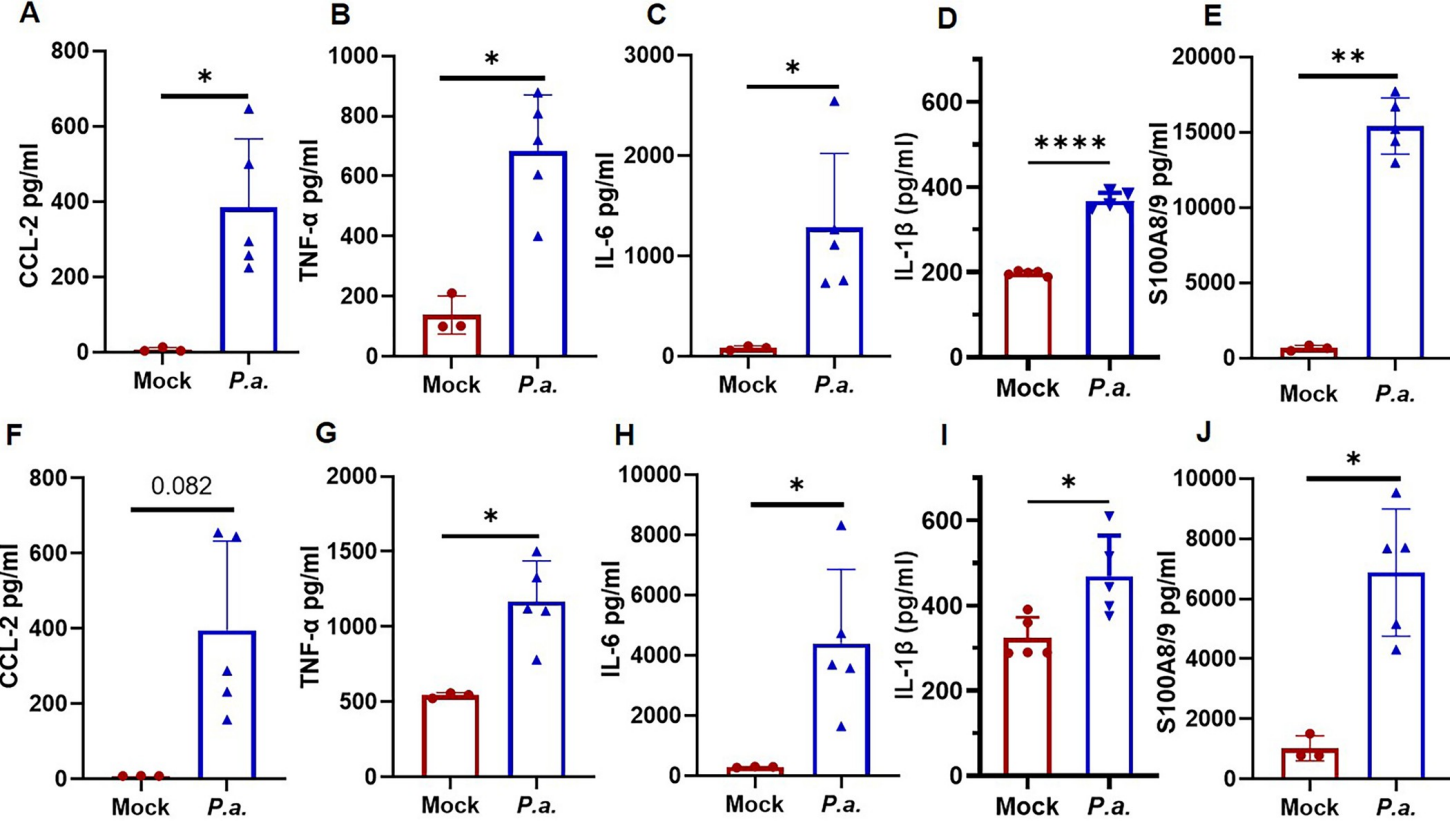
*P*. *aeruginosa infection* increases inflammatory cytokine levels in the BAL and serum. Cytokine and chemokine levels in BALF and serum of control mock uninfected (N = 3) and *P*.*a*. infected mice (N = 5) at 48 post infection were determined by ELISA. Graphs shown in top panels are cytokines present in BALF, (**A**) CCL2, (**B)**) TNF-α, (**C**) IL-6, (**D**) IL-1β, and (**E**) S100A8/9. Bottom panel graphs are serum cytokine levels, (**F**) CCl2, (**G**) TNF-α, (**H**) IL-6, (I) IL-1β and (**J**) S100A8/9. Data shown are a representative of 3 independent experiments, (mean ± SD; *p<0.*05;* **p<0.*005)*.

To test the possibility that increased *P*.*a*. burden in the lung leads to enhanced cytokine levels both locally and systemically, we measured key chemokine and cytokine levels in the BALF and serum. Indeed, *P*.*a*. infection markedly increased CCL2 chemokine levels in the BALF (Fig 5A). We also observed increased level of TNF-α, IL-6 and IL-1β (Fig 5B, 5C and 5D). CCL2 is a major player in the recruitment of immune cells at site of infections, while TNF-α, IL-6 and IL-1β are proinflammatory cytokines. S100A8/A9, also a potent chemotactic agent was significantly increased in the BALF during *P*.*a*. infection (Fig 5E). To correlate these findings with systemic inflammation, we observed similar increases in the levels of CCL2, TNF-α, IL-6, IL-1β and S100A8/A9 in the serum of infected mice (Fig 5F–5J).

### S100A8/A9 restricts bacterial burden within the lungs and partially protects heart function

**Fig 6:**
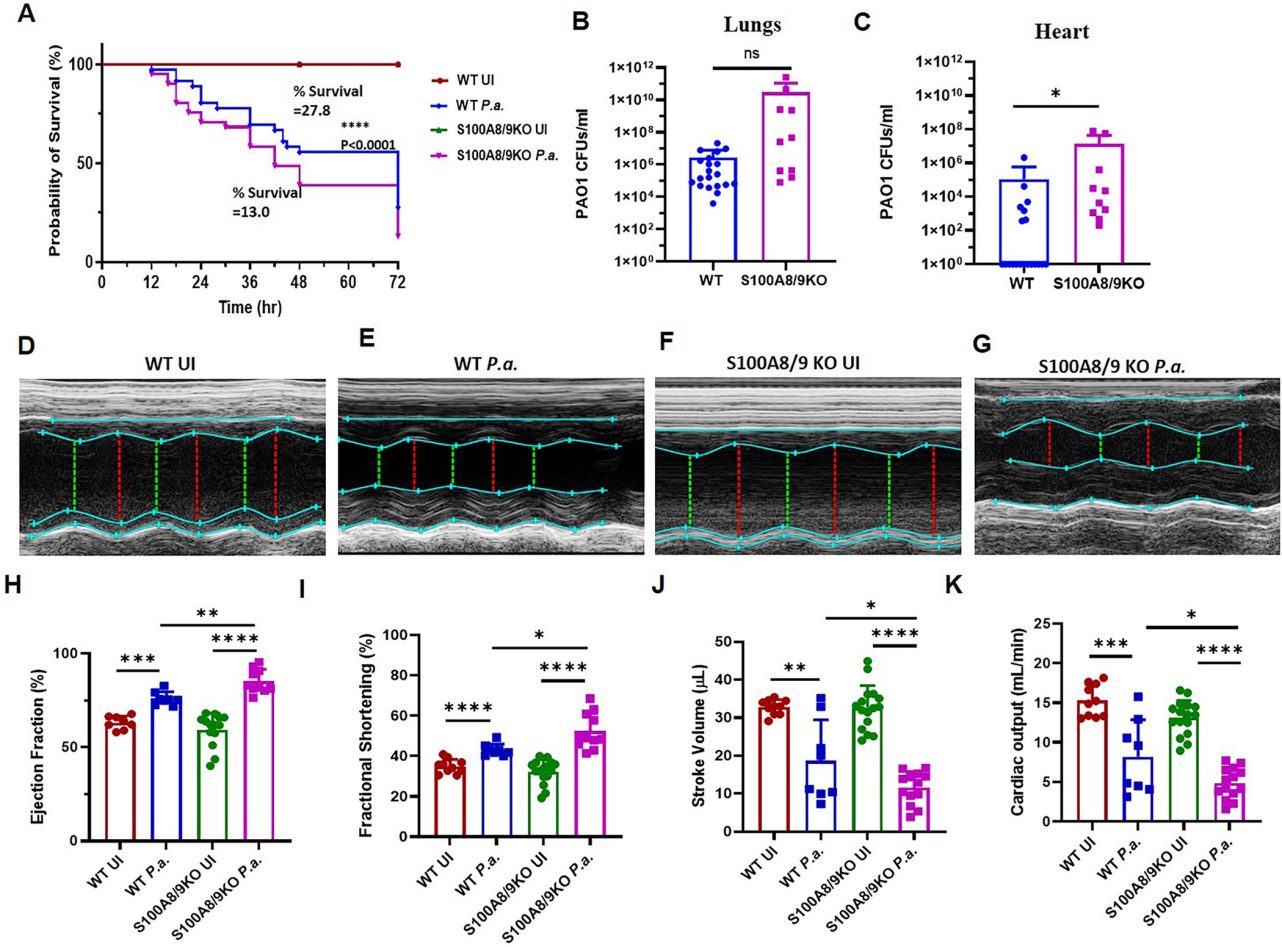
S100A8/9 deficiency increases the bacterial burden in the lungs and causes severe cardiac dysfunction, and mortality. Wild type and S100A8/9 knockout mice (KO) were intranasally infected with sequential doses of *P*.*a*. as described in Fig 1 and survival of the mice were monitored for 72 hours. (**A**) Percent survival was calculated from uninfected control mice. Data shown are the cumulative from 5 experiments (n = 35 mice) mean ± SD (***p<0.*0005*). At the end of the study period, surviving mice were euthanized, lungs (**B)** and hearts (**C**) were harvested and homogenized for CFU assays. Graphs shown are cumulative data from 3 independent experiments (WT = 19 mice; S100A8/9KO = 10 mice), mean ± SD; ns = nonsignificant; (*p<0.*05*). To assess heart function *in vivo*, 2D-echocardiography (Vevo 2100, Visualsonics) was performed with control mock uninfected and *P*.*a*. infected wild type and S100A8/9 KO mice at 48 post infection. Representative images of cardiac patches from wild type uninfected control (**D**), wild type *P*.*a*. infected (**E**), S100A8/9-KO uninfected control (**F**) and S100A8/9-KO *P*.*a*. infected (**G**) mice. At least 3 M-mode echocardiographic measurements from each mouse were used to determine the % ejection fraction (**H**), % fractional shortening (**I**), stroke volume (**J**) and cardiac output (**K**). Data shown are the mean ± SD of cumulative data from three independent experiments (Wt, N = 8; S100A8/9-KO, N = 12), ***p<0.0005; ****p<0.*0005*.

Previous studies reported that more than 40% of all cytoplasmic proteins in neutrophils is S100A8/A9 [25], which is readily and rapidly deployed by neutrophils when they encounter infectious agents. As expected, we found an increased levels of S100A8/A9 in the BALF and serum of *P*.*a*. infected mice (Fig 5F and 5J). S100A8/A9 can suppress bacterial growth by chelating metal ions required for bacterial metabolism (25). In addition, the S100A8/A9 complex interacts with pattern recognition receptors such as TLR4 and RAGE on innate immune cells to increase the production of various inflammatory cytokines, and amplify inflammation [47]. Since, we found a robust increase in S100A8/A9 levels both in lungs and blood (Fig 5), we hypothesized that S100A8/A9 may play vital role in restricting *P*.*a*. growth and enhancing the inflammatory response. To test this, we performed *in vivo P*.*a*. infection studies using a global *S100a9* knockout (deficient of both S100A8 and S100A9 proteins) and wild type (WT) mice. *P*.*a*. infection significantly increased mortality of *S100a9* KO mice compared to WT mice (Fig 6A). We found an increased bacterial burden in the lungs of *S100a9* KO mice compared to WT mice (Fig 6B). Interestingly, we also observed increased bacterial burden in the hearts of *S100a9* KO mice compared to WT mice (Fig 6C). Notably, only few WT mice showed bacterial colonization in the hearts (7 out of 36 mice). In contrast, in all *S100a9* KO mice (10 out of 10), *P*.*a*. disseminated into the heart, indicating S100A8/9 plays a critical role in restricting *P.a.* to the lungs during infection. We next examined the cardiac electrical activity and heart function of WT and *S100a9* KO mice during *P*.*a*. infection. The ECG traces shown in S5A and S5B Fig are representative of severely infected mice. Specifically, we found that *P*.*a*. infection caused severe cardiac electrical dysfunction, as evidenced by one or more missing QRS peaks which suggests that these mice are experiencing severe second-degree AV block and cardiac arrhythmia. Furthermore, the RR- and QT-intervals were altered by *P*.*a*. infection in both WT and *S100a9* KO mice. However, there was a significant increase of RR and QT intervals in the *S100a9* KO infected mice compared to WT (S5C and S5D Fig). Also, the heart rates of infected mice were significantly reduced in both WT and *S100a9* mice (S5E Fig). The left ventricular (LV) echocardiogram of representative *P*.*a*. infected WT and *S100a9* KO mice also showed increased LV wall thickness as compared to uninfected WT or *S100a9* KO mice (Fig 6D–6G). As a result, there was a significant decrease in ejection fraction and fractional shortening (Fig 6H and 6I) and a corresponding reduction in stroke volume and cardiac output of the left ventricle during *P*.*a*. infection (Fig 6J and 6K). Interestingly, *S100a9* deficiency further enhanced the severity of left ventricular dysfunction as evidenced by a significant decrease in ejection fraction and fractional shortening (Fig 6H and 6I) compared to infected WT mice. Similarly, we observed a significant decrease in stroke volume and cardiac output (Fig 6J and 6K). Together, our data strongly indicate that S100A8/A9 is required for restricting the bacterial burden in the lungs and in the absence of S100A8/A9 there is increased dissemination of bacteria to the heart resulting in severe AV block, cardiac arrhythmia and left ventricular dysfunction.

### S100A8/A9 deficiency increases inflammatory macrophage accumulation in the lungs during *P*.*a*. infection

We next examined whether S100A8/A9 deletion alters the recruitment of neutrophils and other inflammatory cells in the lungs. We harvested the BALF from WT and *S100a9* KO mice with and without infection and used flow cytometry to analyze cell phenotypes. The gating strategy to identify the different immune cell populations is provided in S6A and S6B Fig. Though S100A8/A9 plays a critical role in the immune response at sites of infection, our data revealed that S100A8/A9 deficiency did not impact the number of BAL cells and CD45^+^ cells in the BALF (S6C and S6D Fig). Notably, the infiltration of CD11b^+^ cells and neutrophils were also not affected by S100A8/A9 (S6E and S6F Fig). Although, we observed an overall decrease in the alveolar macrophage population in the BALF of both WT and S100A8/A9, there was a significant increase in number of infiltrating inflammatory macrophages (F4/80^+^ CCR2^+^ cells) in *S100a9* KO mice upon infection (S6G and S6H Fig). Furthermore, we observed a trend of increased level of inflammatory cytokines (TNF-α and IL6) and chemokine (CCL2) in the BALF of *S100a9* KO mice compared to WT mice, but the differences were not statistically significant (S7 Fig).

### Pharmacological targeting of S100A8/A9 decreases mortality during early infection by inhibiting S100A8/A9 mediated immune activation

**Fig 7:**
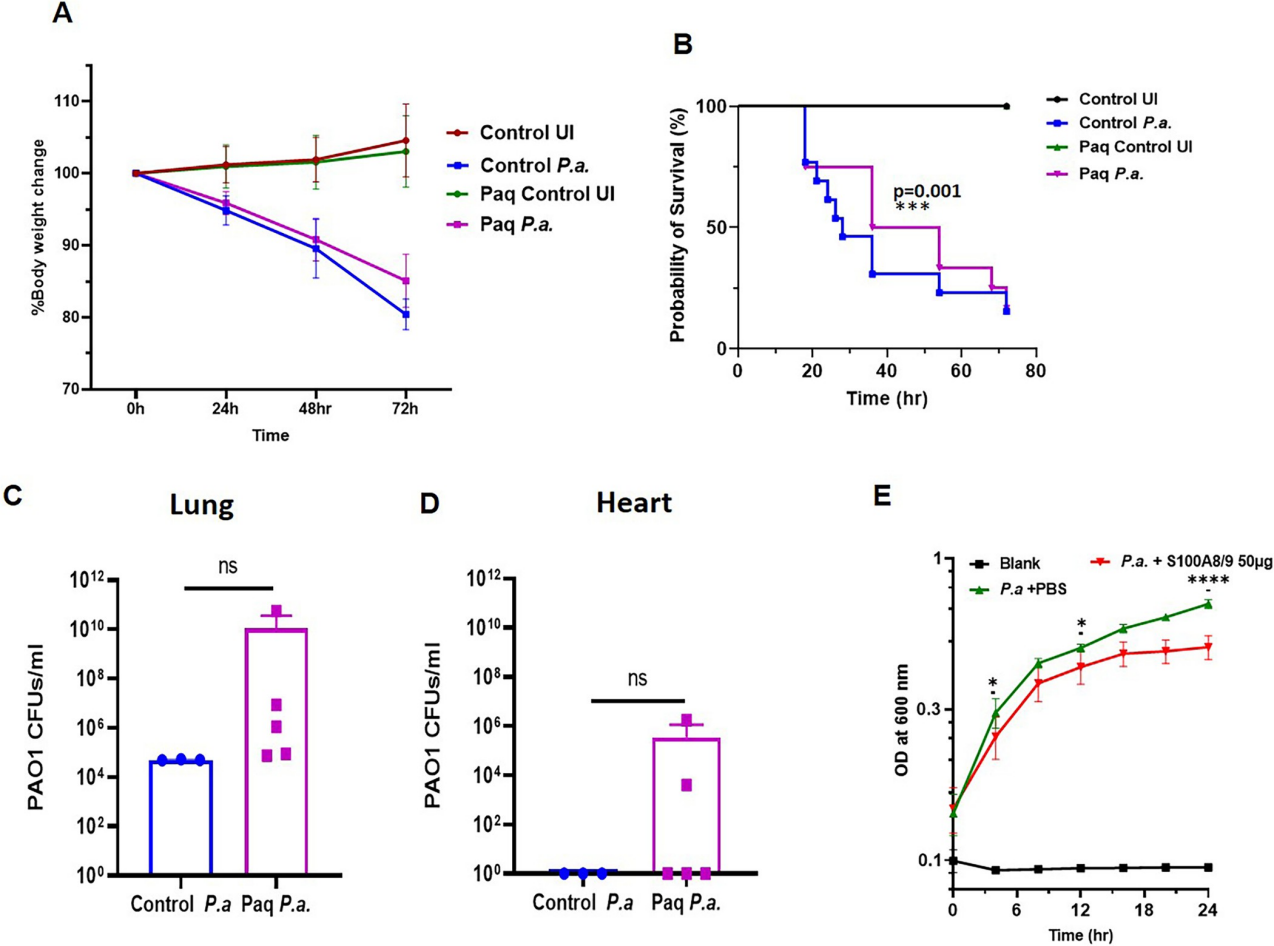
S100A8/9 inhibition partially protects mice from *P*.*a*. induced death. C57BL/6 mice were pre-treated with paquinimod (10mg/kg) for 10 days or treated with vehicle control and then infected with two doses of *P*.*a*. as described in Fig 1. Body weight and survival of the mice were monitored for 72 hours. (**A**) Percent weight loss and (**B**) survival rate was calculated every 24h following infection. Data shown are cumulative data from two independent experiments (N = 13) mean ± SD ***p<0.*0005*. At the end of the study period the surviving mice were euthanized (control *P*.*a*. n = 3 and paquinimod *P*.*a*. n = 5), lungs (C) and hearts (D) were harvested, homogenized, and bacterial burden was quantified. (**E**) *P*.*a*. growth in AP medium pre-treated with recombinant S100A8/9 for 30 minutes and then inoculated with *P*.*a*. Growth of *P*.*a*. overtime was monitored by measuring the O.D. @ 640nm. Graph shown is a representative of 3 independent experiments performed in triplicate wells, mean ± SD; *p<0.*05;* ***p<0.*0005)*.

As mentioned earlier S100A8/A9 provides nutritional immunity to the host by sequestrating essential metal ions [26] and also by involving TLR4 and RAGE pathways, which mediates the host inflammatory response to control *P*.*a*. infection [29,48]. Consistent with this notion, we found that *S100a9* KO mice were not able to control *P*.*a*. infection (Fig 6). To test whether lack of activation of the immune response by S100A8/A9 is the main reason for the enhanced bacterial growth in *S100a9* KO mice, we used paquinimod (Paq), a specific inhibitor of S100A8/A9 binding to the TLR4 and RAGE receptors [49]. Mice were treated with Paq or vehicle for 10 days and then intranasally infected with *P*.*a*. There was significant weight loss during early stages in both control and Paq-treated mice that were infected with *P*.*a*. (Fig 7A). However, the Paq-treated mice showed significantly lower weight loss at 72 hours. *P*.*a*. significantly decreased survival of control mice, but in contrast Paq pre-treatment partially protected the mice from *P*.*a*. infection at 48-hour post-infection. Interestingly, the Paq-mediated protection was lost at 72-hour post-infection (Fig 7B). Paq pre-treatment slightly increased the bacterial load in the lungs (Fig 7C) suggesting that S100A8/A9 mediated activation of TLR4/RAGE signaling is required for efficient bacterial killing. Interestingly, Paq treatment led to increased bacterial dissemination into the heart (Fig 7D). Next, to test whether intranasal delivery of S100A/9 inhibitor Paq protects the mice from *P*.*a*. infection, we delivered Paq to *P*.*a*. infected mice via intranasal route at 6-hour post infection and monitored mouse survival, and cardiac electrical activity. Our results revealed that Paq treatment slightly enhanced the survival of mice with increased trend of bacterial burden in the lungs, however the survival rate was not significantly different (S8A and S8B Fig). Also, we examined whether the Paq treatment reverse the *P*.*a*. induced cardiac electrical dysfunction. Our results demonstrated that the Paq treatment worsened the cardiac electrical conduction as evidenced by a decrease in heart rate, similar to vehicle treated P.a. infected mice (S8C Fig) and an increased the PR intervals, PR intervals and Qt intervals (S8D–S8F Fig), suggesting that the S100A8/9 mediated host immune response is required for efficient *P*.*a*. killing. To test whether S100A8/A9 has direct effect on bacterial growth, we exposed *P*.*a*. to recombinant S100A8/A9 and monitored growth over the period of 24 hours. Indeed, exposure of *P*.*a*. with S100A8/A9 significantly reduced bacterial growth (Fig 7E). These data strongly suggest that increased neutrophil recruitment and robust release of S100A8/A9 at the local environment controls bacterial growth and provides protection. However uncontrolled production of S100A8/A9 may activate myeloid cells *via* TLR4/RAGE leading to hyper-activation of immune cells and increased production of inflammatory mediators that can cause tissue damage and increase the mortality.

## Discussion

Pneumonia is a leading cause of death in the United States for critically ill patients, and *P*.*a*. is one of the most common causes of these infections [7,18,50]. Bacteremia and sepsis frequently occur, and cardiac dysfunction contributes significantly to the pathology and death in these patients [51]. *P*.*a*. produces a variety of virulence factors, including exotoxins and proteases which damage host tissue [52]. Previous studies demonstrate that *P*.*a*. can damage the lung epithelium via the type 3 secretion system (T3SS), which can then lead to dissemination into the bloodstream resulting in septic shock and cardiac dysfunction [53]. However, abrupt cardiac failure in pneumonia patients has also been observed in the absence of septic shock [54]. Therefore, it remains unclear if bacteremia is mandatory for cardiac failure during pneumonia. In this study, significant cardiac electrical and left ventricular dysfunction occurred despite limited dissemination of bacteria out of the lungs (Figs 1 and 2). One potential explanation for this is that *P*.*a*. releases components, which, along with other host inflammatory mediators, may enter the bloodstream leading to heart damage. We predict that release of bacterial products into the bloodstream may be an important mechanism for pathogenesis during sepsis.

There is growing evidence suggesting that infection with other bacterial species can also lead to cardiac dysfunction. Recently, Brown *et al*., demonstrated that *Streptococcus pneumoniae* infection causes aberrant cardiac electrophysiology due to the translocation of bacteria into the myocardium. The release of toxins results in the formation of microlesions filled with extracellular *S*. *pneumoniae*. However, limiting numbers of immune cells were observed at the site of these infections [55]. In an opposing manner, *Staphylococcus aureus*-mediated cardiac lesions results in an abundance of immune cells at the infection site [56]. We have also previously reported that *Francisella novicida* infection in mice causes cardiac dysfunction with altered electrophysiology. Like *S*. *aureus*, *F*. *novicida* translocated into the heart resulting in micro lesions filled with immune- and apoptotic cells [35]. *Mycobacterium tuberculosis* can also cause pericarditis, endocarditis, and myocarditis leading to sudden death in tuberculosis patients [57]. Taken together, these studies indicate that the mechanism by which bacterial infections contribute to cardiac dysfunction likely varies between bacterial species. In the current study, we report that *P*.*a*. infection is also capable of altering normal cardiac electrical activity and heart function.

Cardiac fibrosis is associated with many forms of heart disease and commonly occurs because of septic shock. Cardiac fibroblasts are important for maintaining homeostasis in the heart. However, following injury, the accumulation of the extracellular matrix components produced by fibroblasts may lead to structural deformation, chamber dilation, and hypertrophy leading to poor function [58]. In addition to fibroblasts, an abundance of inflammatory macrophages accumulates in the heart tissue following severe cases of septic shock [59]. There are two major classes of cardiac macrophages: tissue resident and blood-derived infiltrating macrophages [13]. Tissue resident macrophages perform homeostatic functions, and are maintained by self–renewal [60]. Conversely, the infiltrating blood-derived macrophages are inflammatory and important for clearing pathogens [61]. While infiltrating macrophages are indeed critical in host defense, excessive activity of this population could induce collateral tissue damage [62–64]. We found that *P*.*a*. infection resulted in extensive infiltration of myeloid cells (Fig 3). Notably, our data revealed an increased number of neutrophils, monocytes, and macrophages, (particularly MHC-II^high^) in the heart following *P*.*a*. infection. These macrophages expresses both inflammatory (CCR2 and IL-1β) and anti-inflammatory (IL-10 and TGF-β) markers (S3 Fig), indicating that the cardiac macrophages are heterogeneous. Together, this data suggests inflammatory macrophages contribute significantly to the cardiac damage that occurs during *P*.*a*. pneumonia.

It is well-known that the immune response during pneumonia involves the simultaneous recruitment of myeloid cells and the secretion of inflammatory mediators [65]. In addition, an increased number of neutrophils in the lungs and increased levels of pro-inflammatory cytokines such as IL-1β and TNFα in the serum is commonly observed during sepsis and, is associated with cardiac failure and death [35]. The present study provides a novel view as to how pneumonia causes alveolar damage and affects heart function. *P*.*a*. infection enhances the infiltration of myeloid cells into the lungs, specifically neutrophils, resulting in release of inflammatory cytokines and S100A8/A9. Alveolar macrophages (AMs) are the first phagocytic cells that encounter invaded pathogens and efficiently clear the pathogens by secreting complement factors, reactive oxygen species, and inflammatory cytokines [66]. Also, these AMs play a critical role in resolving lung injury [67]. *P*.*a*. infection enhances AM apoptosis via activation of redox signaling and JNK [68]. Consistent with the earlier studies, we found that *P*.*a*. infection completely depleted the CD11c+ AM population. Additionally, we observed increased levels of CCL2, TNF-α, IL-6, and S100A8/9 in the BALF and serum, suggesting that the recruitment of neutrophils into the alveolar space could be a reason for an increased inflammatory cytokine production and lung damage and ultimately inducing systemic inflammation during *P*.*a*. infection. Together, our data indicates that depletion of AMs, increased recruitment of neutrophils with subsequent release of S100A/A9, and hyper-activation of systemic inflammation are the primary sources for cardiac dysfunction upon *P*.*a*. infection.

During infection, neutrophils are the major source for S100A8/A9, which modulate the inflammatory process via induction of inflammatory cytokines, reactive oxygen species, and nitric oxide [25]. Deletion of S100A8/A9 promotes progression of bacterial pneumonia [69] and septic patients showed continuous increase of S100A8/A9 [70]. In addition, the S100A8/A9 proteins have antibacterial activity via metal sequestration [25,27,28]. The excessive production of S100A8/A9 magnifies the inflammatory response via the involvement of TLR4 and RAGE pathways, and ultimately increases the release of inflammatory cytokines by neutrophils, macrophages, which may lead to hyper-activation, systemic inflammation, and organ damage [25,32]. However, the potential role of S100A8/A9 in cardiac dysfunction during *P*.*a*. infection is largely unknown. Herein, we demonstrate that, during *P*.*a*. infection, S100A8/A9 deficiency significantly enhanced mouse morbidity and mortality, and that S100A8A/9 deficiency promotes bacterial dissemination to the heart resulting in severe cardiac dysfunction (Fig 6). This is supported by an increased infiltration of myeloid cells into the lungs and systemic inflammation (S5 Fig). The binding of S100A8/A9 to its receptors TLR4 and RAGE and downstream inflammatory response is inhibited by paquinimod [71]. Therefore, we tested whether blocking S100A8/A9 receptor interaction protects mice from *P*.*a*.*-*induced death. Our results strongly support this as we observed increased survival of mice during early stages of infection but failure to control bacterial growth at later stages. Also, our data revealed that intranasal delivery of Paq in *P*.*a*. infected mice did not protect the mice from *P*.*a*. induced cardiac dysfunction (S8 Fig). Thus, we predict that the early protection is due to an increased bacterial killing by S100A8/A9 and the host immune response, however rapid and uncontrolled growth of *P*.*a*. caused dissemination of bacteria into the heart and other organs and induced the death. Furthermore, our data show that incubation of *P*.*a*. with recombinant S100A8/A9 protein enhanced the bacterial killing (Fig 7). Taken together, release of S100A8A/9 is critical for controlling bacterial growth and mounting an innate immune response, but the release of S100A8/A9 must be tightly controlled both spatially and temporally for efficient control of *P*.*a*. in the lungs.

In conclusion, we demonstrated that pneumonia caused by *P*.*a*. infection can induce cardiac injury independent of dissemination out of the lungs. During infection, we observe an abundance of monocytes, macrophages, and neutrophils in the heart which led to severe cardiac dysfunction. While it remains unclear which *P*.*a*. factors are involved in this process, additional studies will be necessary to identify the precise factors that are released into the bloodstream to promote immune cell recruitment to the heart. Furthermore, we determined that S100A8/A9 released from innate immune cells are the major regulators of the disease pathogenesis. Deletion of S100A8A/9 enhanced mice mortality, suggesting that neutrophil recruitment and release of S100A8/A9 is critical to control *P*.*a*. replication. However, the uncontrolled release of S100A8/A9 aggravates the immune response via TLR4/RAGE receptor, causing tissue damage, systemic inflammation, bacterial dissemination, and cardiac dysfunction. Inhibition of S100A8/A9-mediated immune response and the bactericidal activity of S100A8/A9 transiently protects mice from *P*.*a*. infection. Therefore, our study reveals that *P*.*a*. infection causes cardiac dysfunction by either directly dissemination into the heart or due to S100A8/A9-medated activation of the host immune response.

## Material and methods

### Ethics statement

All animal procedures in this study were approved by The Ohio State University Institutional Animal Care and Use Committee (IACUC) under protocol 2020A00000004.

### Bacterial growth and media

*P*.*a*. cultures were grown overnight in Luria broth without NaCl (LBNS) at 37°C. In all experiments, *P*.*a*. was grown to log phase (0.5–0.8 OD_600_) prior to infection by inoculating 100 μl of overnight culture into LBNS.

### Murine pulmonary infection and bacterial burden

Eight-week-old male and female C57BL/6 mice were anesthetized with isoflurane and administered 1x10^8^ cells of the *P*.*a*. strain PAO1 in 30 μl of saline via intranasal instillation. After 24 hours, mice were inoculated a second time with 1x10^7^ of *P*.*a*. Mock infected mice were treated with 30 μl of sterile saline in the same manner. Some experiments, *P*.*a*. infected mice were treated with tobramycin (30 mg/kg) via intraperitoneal route or paquinimod (12.5 μg/mice in 50μL saline) via intranasal route, at 6 hour post infection. The mice were euthanized 48 hours post-infection (h.p.i.) and the lungs and hearts were harvested. For CFU quantification, tissue was homogenized in PBS and plated on sterile *Pseudomonas* isolation agar (PIA) plates. Serial dilution of the sample was performed in triplicate for the quantification.

### Electrocardiogram (ECG) and echocardiogram

After 48h of *P*.*a*. infection, subsurface Electrocardiogram (ECG) was recorded. *P*.*a*. infected mice and uninfected control mice were anesthetized using 2% isoflurane in oxygen (flow rate 1.0 L/min), which was subsequently lowered to 1% (1.0 L/min) for the duration of the ECG recordings. Mice were placed in the prone position and kept on a heated pad to maintain body temperature. The subcutaneous electrodes for ECG were placed in the lead II configuration and ECGs were recorded for 5 minutes on a Powerlab 4/30 (AD Instruments, Houston, TX) (35). ECG traces were analyzed using LabChart 9 Pro (AD Instruments). To assess cardiac function *in vivo*, 2D-echocardiography (Vevo 2100, Visualsonics) was performed in *P*.*a*. infected or control mice 48h post infection. Mice were anesthetized in an induction chamber at 2% isoflurane in oxygen at a flow rate of 1.0 L/min. Mice were then placed in supine position on a heated stage and hair was removed from the chest using depilatory lotion. Anesthesia was maintained at 1.5% isoflurane for the duration of the experiment. Heart rate was monitored throughout to ensure proper anesthetic dosage. Using a MS-400 transducer, proper anatomical orientation was determined via imaging along the Parasternal long axis (PSLX) of the heart. M-mode images were recorded at the level of the papillary muscles. Images were analyzed to assess ejection fraction, fractional shortening, chamber diameters, left ventricular wall thicknesses and other heart functions.

### Histopathology and Immunohistochemistry

The hearts and lungs of *P*.*a*. infected or uninfected control mice were isolated and washed in cold PBS and then fixed in 10% formalin overnight at 4°C. The organs were then transferred to 20% sucrose solution overnight at 4°C, embedded in paraffin, and sectioned for further analysis. To examine bacterial distribution and dissemination, four chamber view heart sections and lung cross sections were deparrafinized and antigen epitopes were retrieved via heat induced antigen retrieval. Tissue sections were then stained with anti-*P*.*a*. antibody [72] followed by anti-rabbit Alexa Fluor488. The nuclei were then stained with DAPI. Tissue sections were probed with anti CD45 antibody (WistoHiz, NY), and ten random images were taken from each heart (n = 5) and mean fluorescent intensities (MFIs) were calculated by using ImageJ software (NIH, Bethesda, MD). Average MFIs were calculated by multiplying the intensities by area of each image.

### Cardiac cell isolation and flow cytometry analysis

After the mice were euthanized, the hearts were harvested and digested using the Multi Tissue Dissociation Kit and gentleMACS Dissociator (Miltenyi Biotec, Auburn, CA, USA) as per manufacturer’s instructions. Homogenized heart tissue was suspended in 7.5 ml of RPMI containing 20% FBS and passed through 70 μm cell strainers. Then cells were pelleted by centrifugation at 600 x g, further processed with Debris Removal Solution (Miltenyi Biotech), and red blood cells removed via lysis. The cells were counted using an automatic cell counter (NanoEnTek), and dead cells were excluded by staining with trypan blue.

Cells were incubated with mouse FcBlock (BioRad) for 15 minutes and stained with a mixture of flurochrome-conjugated antibodies. Cell populations were analyzed and quantified using a BD Fortessa flow cytometer and compensation, and data analyses were performed using FlowJo Software (FlowJo LCC, OR, USA).

### Cardiac macrophage isolation and RNA extraction

Macrophages were isolated from the heart tissue of infected and control mice by isolating CD11b^+^ cells. Briefly, heart tissue was digested (as above) and incubated with CD11b microbeads for 15 minutes. Cells were then washed and the CD11b^+^ population was isolated using a magnetic column (Miltenyi Biotech, Auburn, CA, USA). The CD11b^+^ cells were suspended in RPMI containing 10% FBS and suspended in tissue culture treated plates. After 2 hours, non-adherent cells were removed, and the macrophage monolayer was lysed to isolate total RNA.

### Bronchoalveolar lavage fluid (BALF) collection from mice and flowcytometry

Bronchoalveolar lavage fluid was collected from the lungs of *P*.*a*. infected and uninfected control mice 48 h.p.i. Mice were euthanized and 20G catheter was inserted into the trachea of mice. Then the BALF was collected by washing (3X) the lungs of infected and control mice with 0.5 ml PBS. Then the BALF was centrifuged at 500g for 10min, the supernatant collected for ELISA, and the cells pellets resuspended in PBS for flow cytometry. For flow cytometry, the cells were enumerated using an automatic cell counter (NanoEnTek), incubated with mouse FcBlock (BioRad) for 15 minutes at room temperature, then washed with PBS. Then cells were immunostained with a mixture of flurochrome-conjugated antibodies (α-CD45, α-CD11b. α-Ly6C, α-Ly6G, α-F4/80, and α-CCR2) for 30 min, at 4°C in the dark. After staining, cells were washed and resuspended in buffer for analysis. Flowcytometry data acquisition was performed on Cytek Aurora using SpectroFlo software (Cytek Biosciences, CA, USA) and data analyses were performed using FlowJo Software (FlowJo LCC, OR, USA).

### Cardiac troponin assay

Serum cardiac-specific troponin-I (cTnI) was determined by using the high-sensitivity mouse cardiac troponin-I ELISA kit from Life Diagnostics, Inc (West Chester, PA). Briefly, serum was isolated from mock infected and *P*.*a*. infected mice, as described earlier, and used for the assay as directed by manufacturer. All assays were performed with 8 mock infected and 9 *P*.*a*. infected animals, and the values obtained were plotted as mean ± SD. Significance was *P*<0.005.

### TUNEL assay

Paraffin-embedded heart sections from sham- and *P*.*a*. infected hearts were deparaffinized with xylene followed by different concentrations of ethanol and processed for antigen retrieval using citrate buffer. Apoptotic cells were stained by the Click-it plus terminal deoxynucleotidyl transferase dUTP nick end labeling (TUNEL) assay for in situ apoptosis detection and Alexa Fluor 488 dye from Molecular Probes Life Technology (Thermo Scientific). Nuclei were stained with Hoechst 33342 solution and examined under confocal microscopy. Ten random images were taken from each heart (n = 5) and mean fluorescent intensities (MFIs) were calculated by using ImageJ software (NIH, Bethesda, MD). Average MFIs were calculated by multiplying the intensities by area of each image.

### Statistical analysis

Statistical analysis was done using R. Statistical comparison of means was performed by Student’s t-test or 2-way ANOVA followed by Tukey post hoc tests, when appropriate. The results are shown as the Mean ± SEM. The threshold for significance was set at p< 0.05.

## Supporting information

S1 Fig *P*. *aeruginosa* infection enhances the cardiac inflammation, apoptosis, and release troponin into the serum. Four-chamber view sections from uninfected and *P*.*a*. infected mice were stained with anti CD45 antibody. (A) CD45 staining of uninfected and infected heart sections by 10× magnification, and the area in the square was enlarged to 40× magnification. (B) Mean fluorescent intensities (MFI) of CD45 staining per μm^2^ indicates infiltration of immune cells in the heart. To determine the apoptosis of heart tissue during P.a. infection, heart sections from uninfected and P.a. infected mice were stained with Click-it plus TUNEL assay kit with Alexa Fluor 488 dye. (C) Representative image from the posterior region of heart sections of control mice (right panel) and *P*.*a*. -infected mice (left panel). (D) Ten random images were taken from each heart (n = 5) and mean fluorescent intensities (MFIs) were calculated by using ImageJ software (NIH, Bethesda, MD). Average MFIs were calculated by multiplying the intensities by area of each image, ***P<0.0005). AF488 indicates Alexa Fluor 488; DAPI, 4’,6-diamidino-2-phenylindole; DIC, differential interference contrast; TUNEL, terminal deoxynucleotidyl transferase dUTP nick end labeling. (E) To assess whether *P*.*a*. infection causes cardiac damage, we harvested serum from mock (n = 6) and *P*.*a*. infected mice (n = 8) and determined the cardiac troponin levels by ELISA. Data shown are the mean ± SD of accumulative data from three independent experiments (N = 8–9), ***P<0.*0005*.
(TIF)

S2 Fig *P*. *aeruginosa* infection causes left ventricular dysfunction. To assess heart function *in vivo*, 2D-echocardiography (Vevo 2100, Visualsonics) was performed in control mock uninfected mice (N = 8) and *P*.*a*. infected mice (N = 11) at 48h post infection. The LVPWd, posterior wall thickness in diastole (**E**); LVPWs, posterior wall thickness in systole (**F**), LVAWd, anterior wall thickness in diastole (**G**) and LVAWs, anterior wall thickness in systole (**H**) were determined from the recorded echocardiographic data. Data shown are the mean ± SD of accumulative data from three independent experiments (N = 8–11), ns-non-significant; *P<0.*05;* **P<0.*005*.
(TIF)

S3 Fig Antibiotic treatment does not rescue cardiac pump dysregulation. C57BL/6 mice were infected with *P*.*a*. and after 6 hours of infection the mice were treated with tobramycin (30mg/kg) in saline or left untreated and continued the antibiotic treatment for every 24hours until endo of the study period. Survival rate was calculated every 24h following infection. (A) Data shown are cumulative data from 10 mice per group mean ± SD ***p<0.*0005*. At the end of the study period the surviving mice were euthanized (control *P*.*a*. n = 3 and tobramycin *P*.*a*. n = 5), lungs (B) and hearts (C) were harvested, homogenized, and bacterial burden was quantified via CFU assay mean ± SD; *p<0.05; ***p<0.*0005*. Heart function was assessed by *in vivo*, 2D-echocardiography (Vevo 2100, Visualsonics) in *P*.*a*. infected control and tobramycin treated mice (N = 3) at 48h post infection. The cardiac output (D), ejection fraction (E), fractional shortening (F) and stroke volume (G) were determined from the recorded echocardiographic data. Data shown are the mean ± SD; *P<0.*05;* **P<0.*005;* ***P<0.*0005*.
(TIF)

S4 Fig *P*. *aeruginosa* infection induces the expression of both inflammatory and anti-inflammatory genes in the cardiac macrophages. After 48h of *P*. *a*. infection or control mock uninfected mice (N = 5), hearts were harvested, and single cell suspensions obtained. CD11b^+^ cells were isolated from cardiac single cell suspension using anti-CD11b microbeads, and total RNA was isolated to determine the expression of CCR2 (**A**), IL-1β (**B**), IL-10 (**C**) and TGF-β (**D**) by qRT-PCR. The graphs shown are cumulative from five animals (mean ± SD; **p<0.005; ***p<0.0005).
(TIF)

S5 Fig S100A8/9 deficiency causes sever cardiac electrical dysfunction during *P*.*a*. infection. Wild type and S100A8/9 knockout mice were intranasally infected with two doses of *P*. *a*. strain PAO1 (as described in Fig 1), and after 48h, cardiac electrical activity was measured by using electrocardiography. (**A**) A representative EKG traces from wild type uninfected control (top panel) and *P*.*a*. infected mice (bottom panel), (**B**) S100A8/9-KO uninfected control (top panel) and *P*.*a*. infected mice (bottom panel). ECG traces were analyzed using Lab Chart 8 Pro (AD Instruments) software and calculated the RR intervals (**C)**, QT intervals (**D**) and heart rate (**E)**. Data shown is a representative of 12 mice from each group, the mean ± SD, *ns-non significant;* ****p<0.*0005*.
(TIF)

S6 Fig *P*. *a*. infection increases neutrophil infiltration and alveolar macrophage apoptosis in the lungs of both wild type and S100A8/9-KO mice. Wild type and S100A8/9-KO were intranasally infected with two doses of *P*. *a*. strain PAO1 (as described in Fig 1). After 48h, mice were euthanized, alveolar lining fluid was obtained by bronchoalveolar lavage (BAL). The myeloid cells were collected by centrifugation of the BAL fluid, stained with CD45, CD11b, F4/80, Ly6C and Ly6G antibodies, and analyzed by multicolor flowcytometry. Representative gating strategies are shown for different cell populations isolated from control uninfected (**A**) and *P*. *a*. infected (**B**) mice. Total BAL cells were counted using automated cell counter (**C)**. Cell population percentages of CD45^+^ cells (**D**), CD11b^+^ cells (**E**), neutrophils (**F**), F4-80^+^ CCR2^+^ macrophages (**G**) and F4-80^+^ macrophages (**H)** were determined. These data are cumulative from 6–9 mice from three independent experiments (mean ± SD, *p<0.*05;* *pP<0.005; *** p<0.0005; ****p<0.00005*)*.
(TIF)

S7 Fig *P*. *a*. infection increases inflammatory cytokine levels in the BALF of S100A8/9-KO mice. Cytokine and chemokine levels in BALF of control uninfected and *P*.*a*. infected wild type and S100A8/9 knockout mice were determined at 48h post infection by ELISA. Graphs shown are levels of TNF-α (**A**), CCL2 (**B**), IL-6 (**C**), and S100A8/9 (**D**) in the BAL fluid. Data shown are cumulative data from 5 mice (mean ± SD; ns-nonsignificant; *p<0.*05)*.
(TIF)

S8 Fig S100A8/9 inhibitor paquinimod treatment does not protect the mice from P.a. induced cardiac electrical dysfunction. C57BL/6 mice were infected with *P*.*a*. and after 6 hours of infection the mice were treated with paquinimod (12.5 μg /mice, in 50 μl saline) or saline alone via intranasal route and monitored the mice survival. (**A**) Data shown are cumulative data from 10 mice per group mean ± SD ***p<0.*0005*. At the end of study period surviving mice were euthanized (control+*P*.*a*. n = 3 and paquinimod+*P*.*a*. n = 3), lungs (**B**) were harvested, homogenized, and bacterial burden was quantified via CFU assay mean ± SD; *p<0.11. Cardiac electrical activity was measured by using electrocardiography and ECG traces were analyzed using Lab Chart 8 Pro (AD Instruments) software and calculated the heart rate (**C**), RR intervals (**D)**, PR intervals (**E**) and QT intervals (**F**). Data shown is an accumulative data from 3 mice/ group, the mean ± SD, *ns-non significant;* **p<0.*005;* ***p<0.0005;****p<0.*00005*.
(TIF)
