# Supplementary figures and images for: Pseudomonas aeruginosa pulmonary infection results in S100A8/A9-dependent cardiac dysfunction
Source: PLoS Pathog. 2023 Aug 25;19(8):e1011573. doi: 10.1371/journal.ppat.1011573 (PMC10484443; doi:10.1371/journal.ppat.1011573)

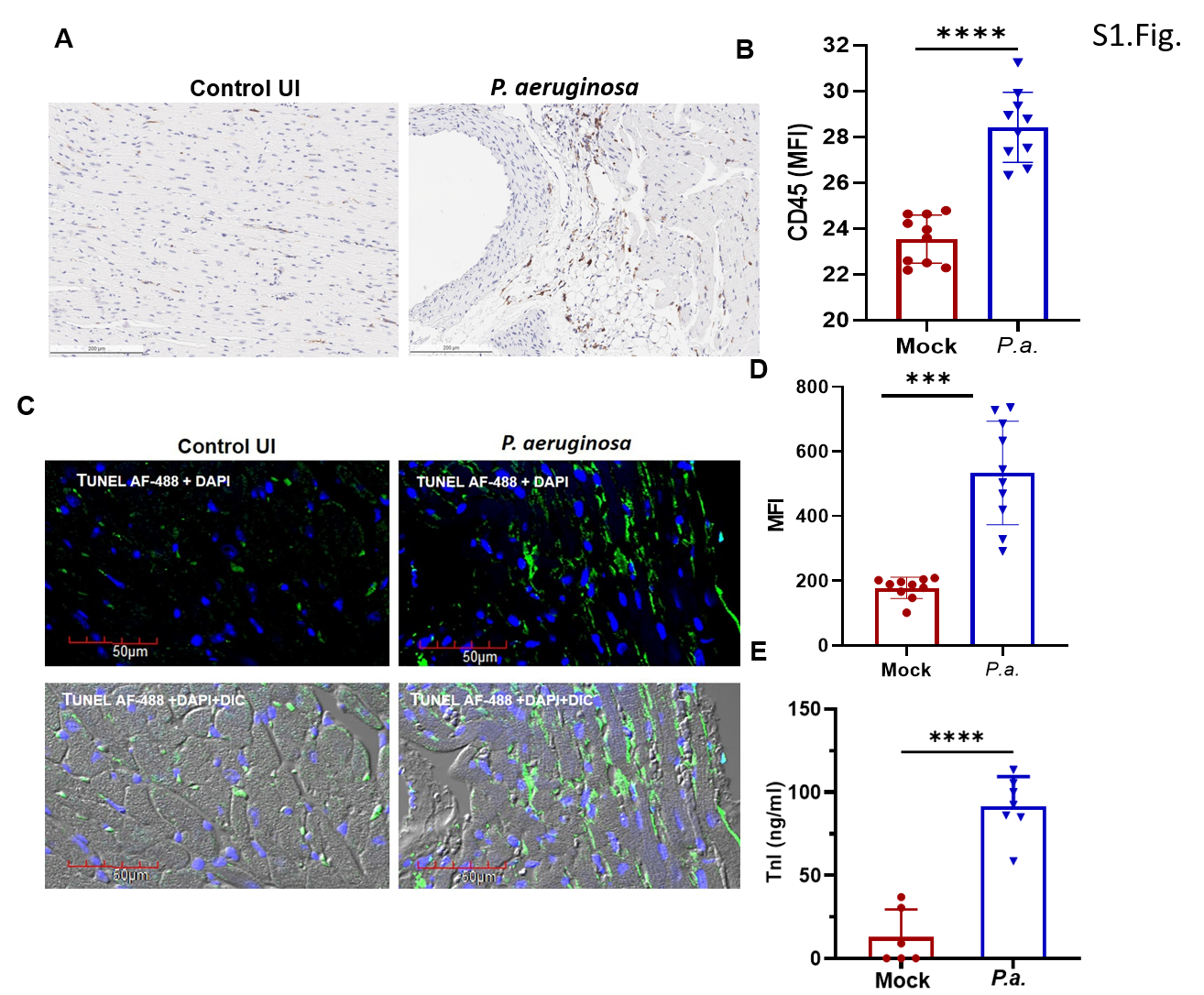

Supplement: S1 Fig — Four-chamber view sections from uninfected and P.a. infected mice were stained with anti CD45 antibody. (A) CD45 staining of uninfected and infected heart sections by 10× magnification, and the area in the square was enlarged to 40× magnification. (B) Mean fluorescent intensities (MFI) of CD45 staining per μm2 indicates infiltration of immune cells in the heart. To determine the apoptosis of heart tissue during P.a. infection, heart sections from uninfected and P.a. infected mice were stained with Click-it plus TUNEL assay kit with Alexa Fluor 488 dye. (C) Representative image from the posterior region of heart sections of control mice (right panel) and P.a. -infected mice (left panel). (D) Ten random images were taken from each heart (n = 5) and mean fluorescent intensities (MFIs) were calculated by using ImageJ software (NIH, Bethesda, MD). Average MFIs were calculated by multiplying the intensities by area of each image, ***P<0.0005). AF488 indicates Alexa Fluor 488; DAPI, 4’,6-diamidino-2-phenylindole; DIC, differential interference contrast; TUNEL, terminal deoxynucleotidyl transferase dUTP nick end labeling. (E) To assess whether P.a. infection causes cardiac damage, we harvested serum from mock (n = 6) and P.a. infected mice (n = 8) and determined the cardiac troponin levels by ELISA. Data shown are the mean ± SD of accumulative data from three independent experiments (N = 8–9), ***P<0.0005. (TIF) [file ppat.1011573.s001.tif]

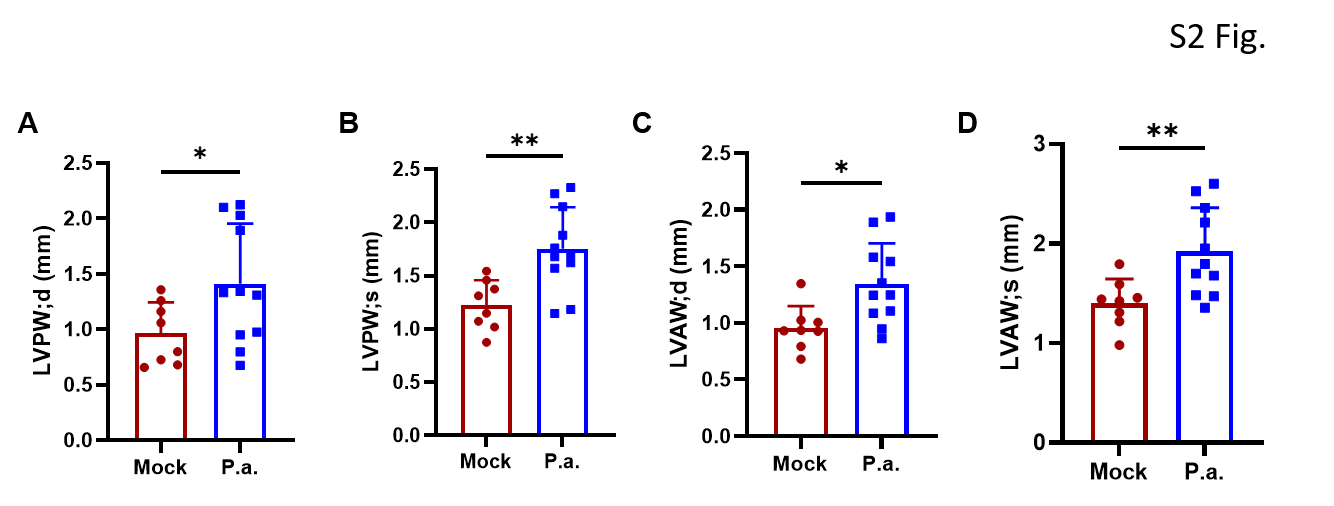

Supplement: S2 Fig — To assess heart function in vivo, 2D-echocardiography (Vevo 2100, Visualsonics) was performed in control mock uninfected mice (N = 8) and P.a. infected mice (N = 11) at 48h post infection. The LVPWd, posterior wall thickness in diastole (E); LVPWs, posterior wall thickness in systole (F), LVAWd, anterior wall thickness in diastole (G) and LVAWs, anterior wall thickness in systole (H) were determined from the recorded echocardiographic data. Data shown are the mean ± SD of accumulative data from three independent experiments (N = 8–11), ns-non-significant; *P<0.05; **P<0.005. (TIF) [file ppat.1011573.s002.tif]

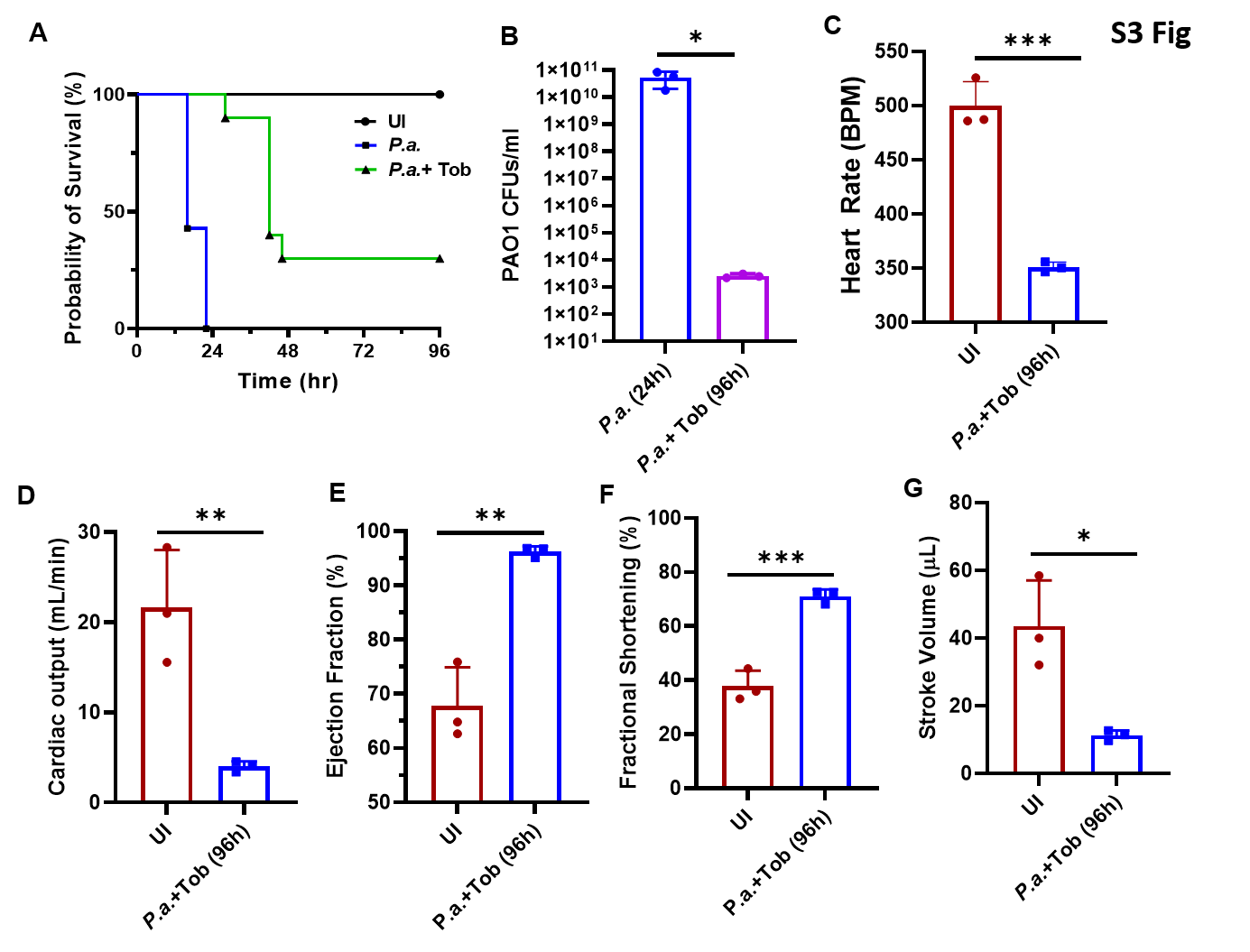

Supplement: S3 Fig — C57BL/6 mice were infected with P.a. and after 6 hours of infection the mice were treated with tobramycin (30mg/kg) in saline or left untreated and continued the antibiotic treatment for every 24hours until endo of the study period. Survival rate was calculated every 24h following infection. (A) Data shown are cumulative data from 10 mice per group mean ± SD ***p<0.0005. At the end of the study period the surviving mice were euthanized (control P.a. n = 3 and tobramycin P.a. n = 5), lungs (B) and hearts (C) were harvested, homogenized, and bacterial burden was quantified via CFU assay mean ± SD; *p<0.05; ***p<0.0005. Heart function was assessed by in vivo, 2D-echocardiography (Vevo 2100, Visualsonics) in P.a. infected control and tobramycin treated mice (N = 3) at 48h post infection. The cardiac output (D), ejection fraction (E), fractional shortening (F) and stroke volume (G) were determined from the recorded echocardiographic data. Data shown are the mean ± SD; *P<0.05; **P<0.005; ***P<0.0005. (TIF) [file ppat.1011573.s003.tif]

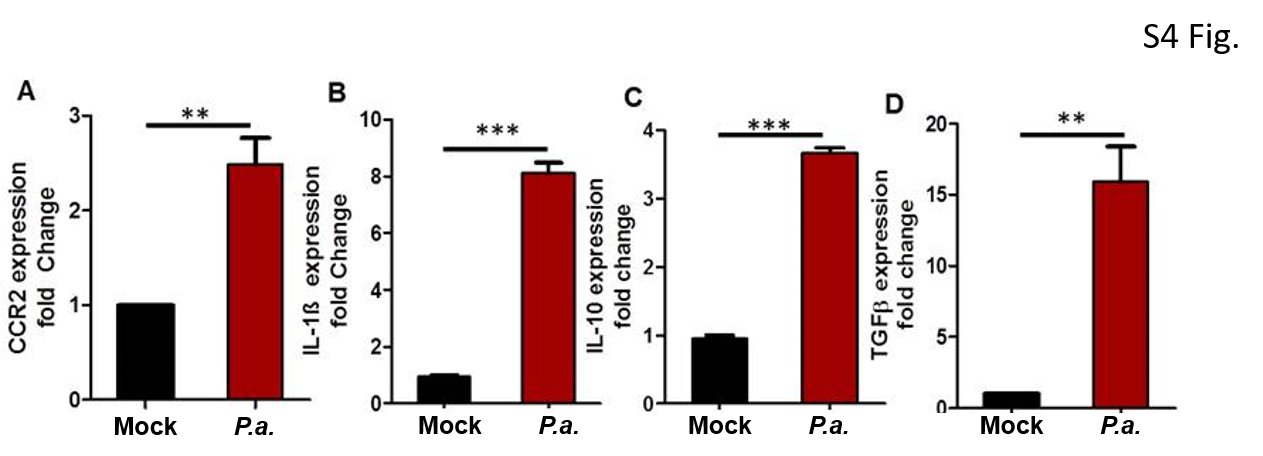

Supplement: S4 Fig — After 48h of P. a. infection or control mock uninfected mice (N = 5), hearts were harvested, and single cell suspensions obtained. CD11b+ cells were isolated from cardiac single cell suspension using anti-CD11b microbeads, and total RNA was isolated to determine the expression of CCR2 (A), IL-1β (B), IL-10 (C) and TGF-β (D) by qRT-PCR. The graphs shown are cumulative from five animals (mean ± SD; **p<0.005; ***p<0.0005). (TIF) [file ppat.1011573.s004.tif]

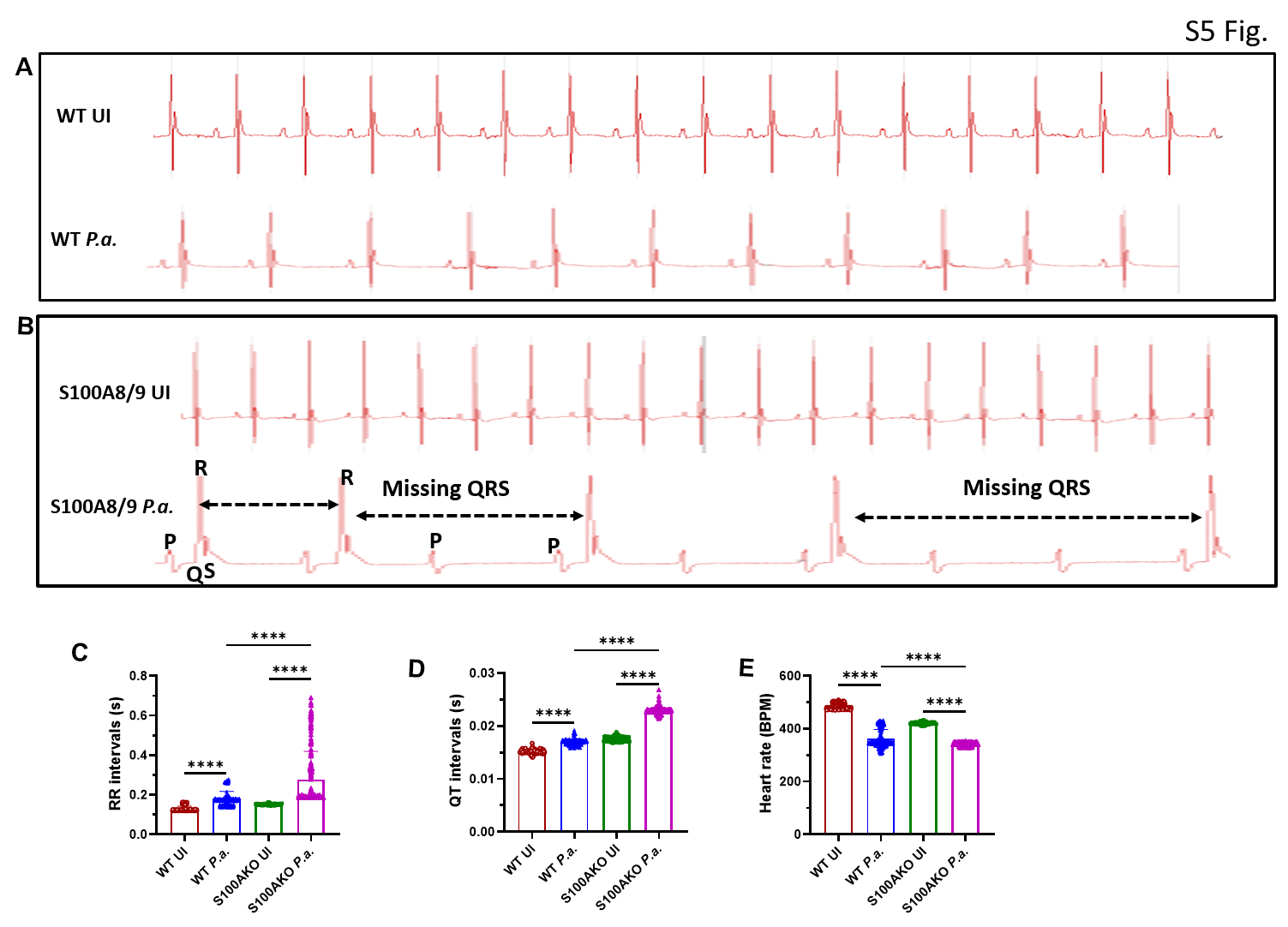

Supplement: S5 Fig — Wild type and S100A8/9 knockout mice were intranasally infected with two doses of P. a. strain PAO1 (as described in Fig 1), and after 48h, cardiac electrical activity was measured by using electrocardiography. (A) A representative EKG traces from wild type uninfected control (top panel) and P.a. infected mice (bottom panel), (B) S100A8/9-KO uninfected control (top panel) and P.a. infected mice (bottom panel). ECG traces were analyzed using Lab Chart 8 Pro (AD Instruments) software and calculated the RR intervals (C), QT intervals (D) and heart rate (E). Data shown is a representative of 12 mice from each group, the mean ± SD, ns-non significant; ****p<0.0005. (TIF) [file ppat.1011573.s005.tif]

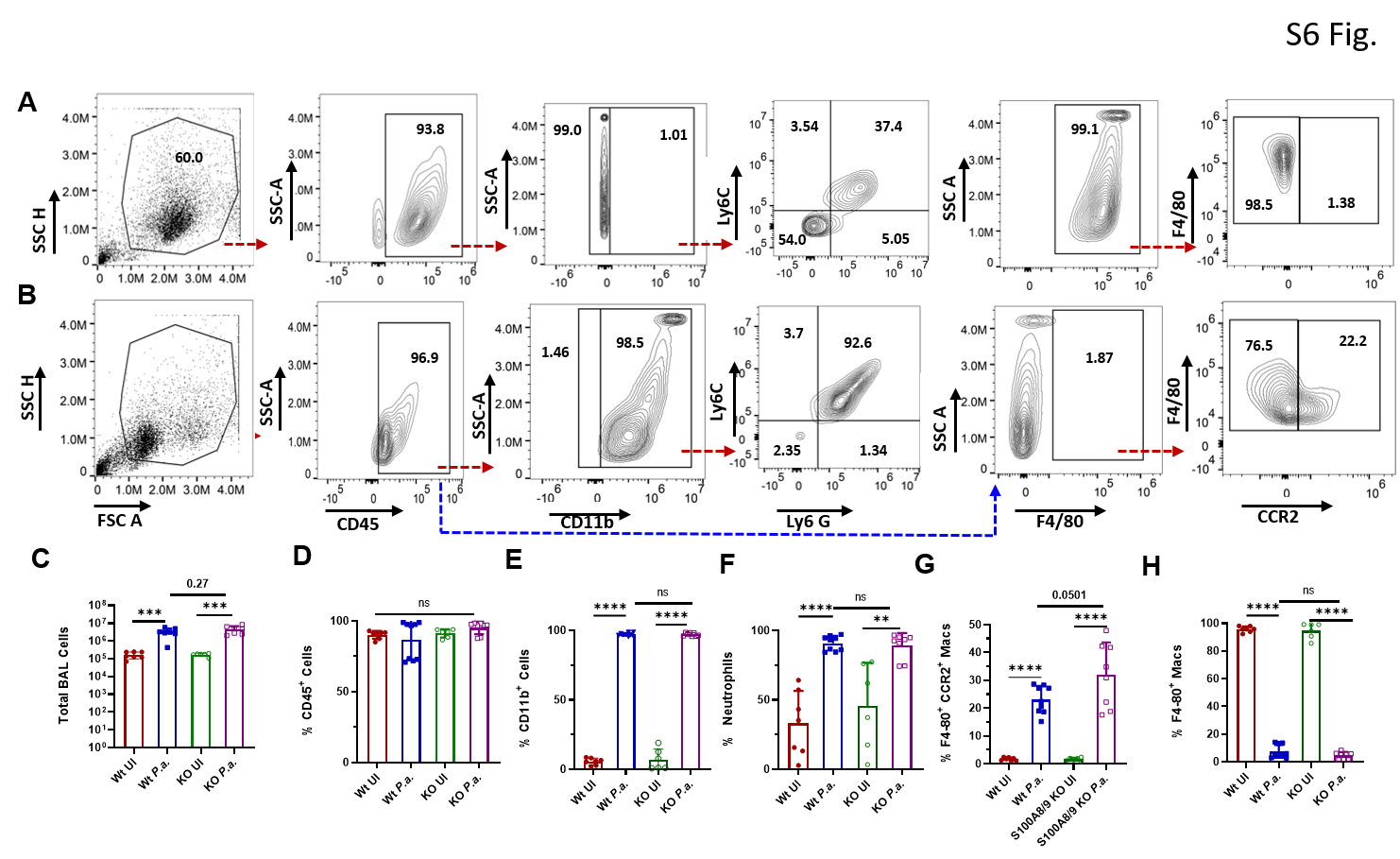

Supplement: S6 Fig — Wild type and S100A8/9-KO were intranasally infected with two doses of P. a. strain PAO1 (as described in Fig 1). After 48h, mice were euthanized, alveolar lining fluid was obtained by bronchoalveolar lavage (BAL). The myeloid cells were collected by centrifugation of the BAL fluid, stained with CD45, CD11b, F4/80, Ly6C and Ly6G antibodies, and analyzed by multicolor flowcytometry. Representative gating strategies are shown for different cell populations isolated from control uninfected (A) and P. a. infected (B) mice. Total BAL cells were counted using automated cell counter (C). Cell population percentages of CD45+ cells (D), CD11b+ cells (E), neutrophils (F), F4-80+ CCR2+ macrophages (G) and F4-80+ macrophages (H) were determined. These data are cumulative from 6–9 mice from three independent experiments (mean ± SD, *p<0.05; *pP<0.005; *** p<0.0005; ****p<0.00005). (TIF) [file ppat.1011573.s006.tif]

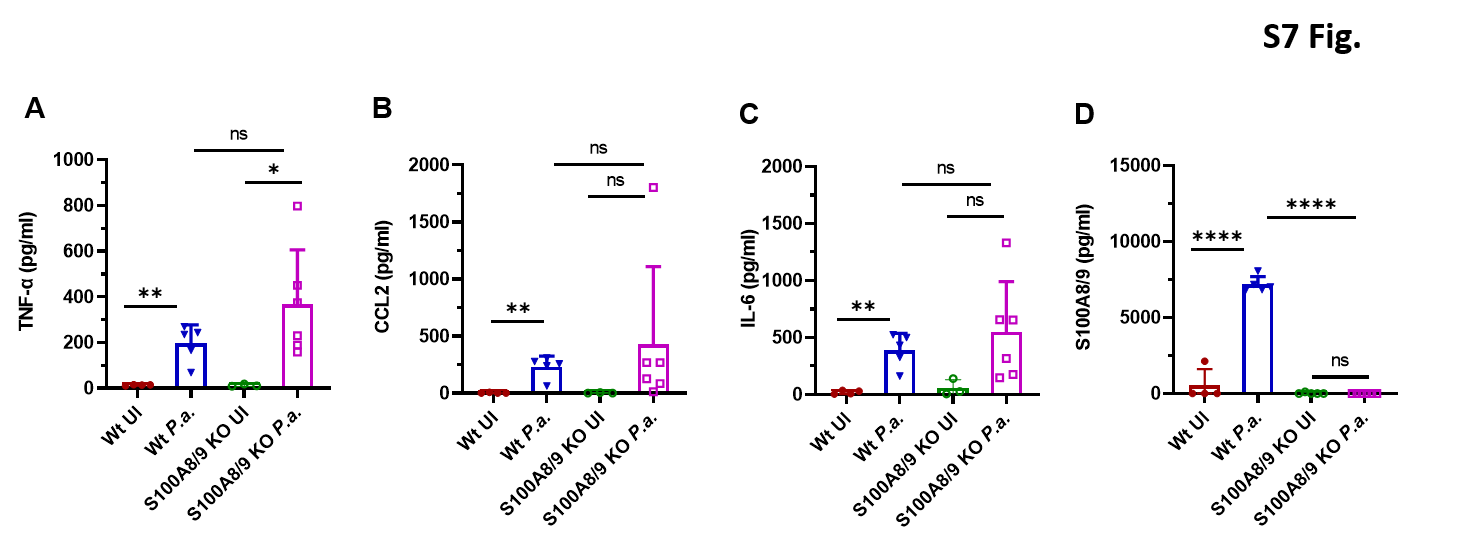

Supplement: S7 Fig — Cytokine and chemokine levels in BALF of control uninfected and P.a. infected wild type and S100A8/9 knockout mice were determined at 48h post infection by ELISA. Graphs shown are levels of TNF-α (A), CCL2 (B), IL-6 (C), and S100A8/9 (D) in the BAL fluid. Data shown are cumulative data from 5 mice (mean ± SD; ns-nonsignificant; *p<0.05). (TIF) [file ppat.1011573.s007.tif]

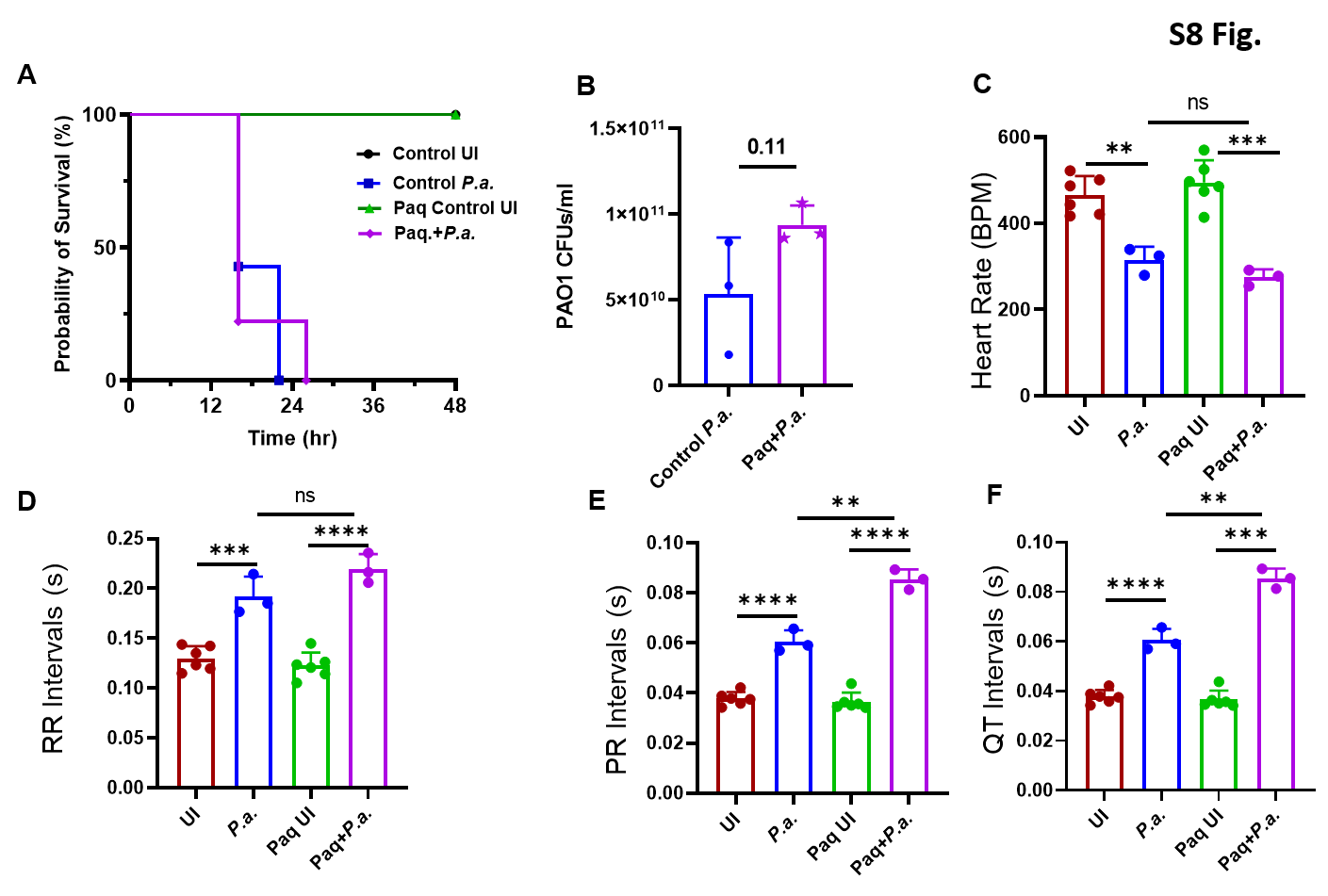

Supplement: S8 Fig — C57BL/6 mice were infected with P.a. and after 6 hours of infection the mice were treated with paquinimod (12.5 μg /mice, in 50 μl saline) or saline alone via intranasal route and monitored the mice survival. (A) Data shown are cumulative data from 10 mice per group mean ± SD ***p<0.0005. At the end of study period surviving mice were euthanized (control+P.a. n = 3 and paquinimod+P.a. n = 3), lungs (B) were harvested, homogenized, and bacterial burden was quantified via CFU assay mean ± SD; *p<0.11. Cardiac electrical activity was measured by using electrocardiography and ECG traces were analyzed using Lab Chart 8 Pro (AD Instruments) software and calculated the heart rate (C), RR intervals (D), PR intervals (E) and QT intervals (F). Data shown is an accumulative data from 3 mice/ group, the mean ± SD, ns-non significant; **p<0.005; ***p<0.0005;****p<0.00005. (TIF) [file ppat.1011573.s008.tif]
